# Antibacterial, Antibiofilm, and Antiadhesive Properties of Different Quaternized Chitosan Derivatives

**DOI:** 10.3390/ijms20246297

**Published:** 2019-12-13

**Authors:** Anna Maria Piras, Semih Esin, Arianna Benedetti, Giuseppantonio Maisetta, Angela Fabiano, Ylenia Zambito, Giovanna Batoni

**Affiliations:** 1 Department of Pharmacy, University of Pisa, 56126 Pisa, Italy; angela.fabiano@unipi.it (A.F.); ylenia.zambito@unipi.it (Y.Z.); 2 Department of Translational Research and New technologies in Medicine and Surgery, University of Pisa, 56126 Pisa, Italy; semih.esin@med.unipi.it (S.E.); a.benedetti16@studenti.unipi.it (A.B.); gmaisetta@biomed.unipi.it (G.M.); 3 Interdepartmental Research Center Nutraceuticals and Food for Health, University of Pisa, 56126 Pisa, Italy

**Keywords:** chitosan, quaternized chitosan, chitosan derivatives, bacteria adhesion, antibiofilm, functionalized titanium, implant-associated infection

## Abstract

In the era of antimicrobial resistance, the identification of new antimicrobials is a research priority at the global level. In this regard, the attention towards functional antimicrobial polymers, with biomedical/pharmaceutical grade, and exerting anti-infective properties has recently grown. The aim of this study was to evaluate the antibacterial, antibiofilm, and antiadhesive properties of a number of quaternized chitosan derivatives that have displayed significant muco-adhesive properties and wound healing promotion features in previous studies. Low (QAL) and high (QAH) molecular weight quaternized chitosan derivatives were synthetized and further modified with thiol moieties or pendant cyclodextrin, and their antibacterial activity evaluated as minimal inhibitory concentrations (MIC) and minimal bactericidal concentrations (MBC). The ability of the derivatives to prevent biofilm formation was assessed by crystal violet staining. Both QAL and QAH derivatives exerted a bactericidal and/or inhibitory activity on the growth of *P. aeruginosa* and *S. epidermidis*. The same compounds also showed marked dose-dependent anti-biofilm activity. Furthermore, the high molecular weight derivative (QAH) was used to functionalize titanium plates. The successful functionalization, demonstrated by electron microscopy, was able to partially inhibit the adhesion of *S. epidermidis* at 6 h of incubation. The shown ability of the chitosan derivatives tested to both inhibit bacterial growth and/or biofilm formation of clinically relevant bacterial species reveals their potential as multifunctional molecules against bacterial infections.

## 1. Introduction

Nowadays, the rapid and worldwide spread of antimicrobial resistance represents one of the major threats to public health. In 2014, the World Health Organization estimated that by 2050, in a worst-case scenario, deaths due to untreatable infections might exceed predicted cancer deaths by 20% leading to an annual number of deaths of 10 million. Despite the dramatic rise in resistance toward conventional antibiotics, the development and approval of new anti-infectious agents have declined dramatically [1]. Scientific barriers to drug discovery and limited financial returns have discouraged the major pharmaceutical companies to invest in the research and development of new anti-infective, and nearly all the new antibiotics brought to the market in the last 25 years are variations of existing drugs rather than novel classes of molecules [2].

To further complicate the picture of antimicrobial resistance is the problem of biofilms, complex communities of microorganisms living attached to a substrate and enclosed in a self-produced extracellular matrix consisting of polysaccharides, proteins and extracellular DNA [3]. It is well recognized that biofilms are involved in a large proportion of human infections and that biofilm-forming bacteria are extremely tolerant to antimicrobial drugs [4]. Multiple biofilm-specific mechanisms contribute to the high level of antibiotic tolerance of biofilms including the barrier-effect played by the extracellular matrix and the low metabolic activity of biofilm cells that render them extremely refractory to antibiotics that target active cell process (e.g., cell wall, protein or nucleic acid synthesis) [5]. Among biofilm-associated infections, those related to the use of medical devices (e.g., central venous or urinary catheters, orthopedic implants, heart valves, endotracheal tubes, dental implants) are particularly relevant [6,7]. Indeed, worldwide increase in life expectancy and the advances in medical technology have led to a greater demand for medical implants and a rising number of implant-associated infections that account for over half of all health-care associated infections. The list of bacterial species causing device-associated infection is quite heterogeneous, but most frequently it includes the Gram-positive staphlylococci (both *S. aureus* and *S. epidermidis*), and the Gram-negative *Pseudomonas aeruginosa*. This latter is an environmental opportunistic bacterium recently classified as a priority pathogen for the research and development of novel antimicrobials due to its increasing antibiotic resistance and its relevance in health care-associated infections [8].

Due to the limited number of available antibiotics, and the similarities in the activity spectrum and mode of action of the existing ones, there is an unmet need to identify new and non-conventional anti-infective therapies able to target not only planktonic bacteria, but also bacteria growing as biofilms. In this regard, the attention towards functional polymers of biomedical/pharmaceutical grade, having anti-infective properties, has recently grown. Since their antimicrobial activity does not generally involve the mechanisms of action of current low molecular weight antibiotics, antimicrobial polymers have the potential to act against microorganisms resistant to conventional drugs. Thus, their application as unconventional antimicrobial agents, may benefit the treatment of multidrug resistant infections, and serve as local delivery systems imparting antibacterial synergism with the loaded cargo or contributing to the overall antibacterial activity of the device/medicine [9]. Among those polymers, chitosan (Ch), a polysaccharide obtained by de-acetylation of chitin, a component of the exoskeleton of crustaceans, exerts a strong antimicrobial activity on a wide variety of microorganisms, such as bacteria, fungi and viruses [10,11,12]. However, its physical-chemical features, such as deacetylation degree, molecular weight (MW) and poor water solubility at physiological pHs, are reported to greatly affect its antimicrobial activity which is mostly limited to pH values below six. The experimental setting used for susceptibility assessment seems also to play a role when evaluating the antimicrobial activity of Ch in vitro as unfavorable interactions of Ch with medium components (e.g., metal ions) may occur, reducing its antimicrobial potency [10]. In this regard, the broth micro-dilution technique has been proposed as the more suitable as opposed to agar-based methods [10]. To overcome the water solubility limitation and/or improve the antimicrobial activity of Ch, several N-modified and/or O-modified Ch derivatives have been described in literature, including quaternized (e.g., trimethylammonium and hydroxipropyltrimethylammonium), phosphorylated, and acyl thiourea derivatives, sulfonated or alkyl sulfonated Ch, and N-alkyl or N-benzyl Ch derivatives [11]. Among those mentioned, quaternary ammonium polymers obtained by different synthetic pathways have shown antimicrobial activity, highlighting their possible application as unconventional antimicrobial agents [12].

In the present study, various Ch quaternary derivatives of different MW have been synthetized and their antibacterial, antibiofilm and antiadhesive properties tested against two medically relevant bacterial species. For the evaluation of the antiadhesive properties, their covalent grafting to titanium surface has been performed.

**Scheme 1:**
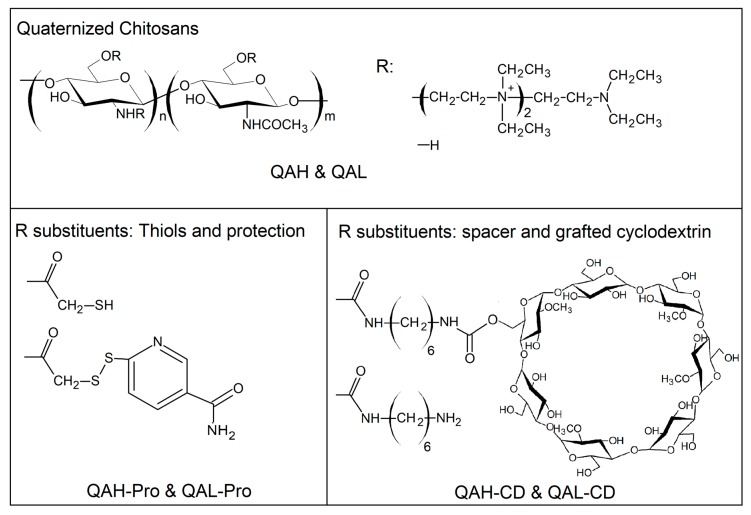
Representative structure of the quaternized Ch-derivatives QAH and QAL further functionalized with either pendant thiols protected with 6-mercaptonicotinamide (QAH-Pro and QAL-Pro) or pendant methylated cyclodextrins (QAH-CD and QAL-CD.

In particular, the attention was focused on quaternary ammonium-chitosans obtained by reaction with 2-diethylaminoethyl chloride (DEAE). The degree of quaternarization of such derivatives (Scheme 1) can be modulated in terms of quaternary chain length and percentage of substitution over chitosans repeating saccharides [13].

The described derivatives (Table 1) differ in MW values (coded QAH and QAL for high and low MW, respectively) but have a similar high degree of substitution (over 80%) and short chains containing a number n of adjacent quaternary ammonium groups (*n* = 2). Their mucus-adhesive capability as well as their wound healing promotion and ocular/intestinal permeation enhancement have already been assessed [13,14,15]. In addition, in a previous work we found that multifunctional quaternary derivatives further derivatized with thiol moieties had improved wound healing features [15] and on this basis, similarly structured, more mucus-adhesive derivatives were obtained with the thiol groups protected from ready oxidation (coded QAH-Pro and QAL-Pro, respectively) [16]. Additionally, it is known that Ch containing cationic or hydrophobic residues can exhibit an enhanced antibacterial/antibiofilm potential [17,18]. Therefore, quaternized Ch grafted with methyl-β-cyclodextrin (coded QAH-CD and QAL-CD, respectively) [19] were also tested, seeing that the cyclic oligosaccharide has a hydrophilic external surface and a hydrophobic internal cavity. All these Ch- derivatives have water solubility irrespective of pH as they all have pendant ammonium quaternary chains. Then, the additional functionalization confers enhanced mucus adhesivity and functional drug complexing capability, for pendant thiol and cyclodextrin respectively. These polymers have been specifically designed and deeply investigated for their application in the pharmaceutical field, highlighting their exploitation either as macromolecular or nanoparticle carrier, but also as thermosensitive hydrogel [20,21].

Being aware of the biopharmaceutical features of such quaternized and functionalized Ch- derivatives, the assessment of their antimicrobial capabilities appears mandatory for their further application in the biomedical field as well as to contribute to a better understanding of the correlation between Ch properties and antimicrobial behavior.

Overall, the results obtained showed ability of most of the Ch-derivatives studied to both inhibit bacterial growth and/or biofilm formation of clinically relevant bacterial species. Partial inhibition of bacterial adhesion to functionalized titanium surfaces was also observed revealing the potential of the Ch-derivatives tested as multifunctional molecules in the anti-infective field.

## 2. Results

### 2.1. Evaluation of MICs and MBCs Values of Quaternized Ch-Derivatives against P. aeruginosa and S. epidermidis

**Figure 1:**
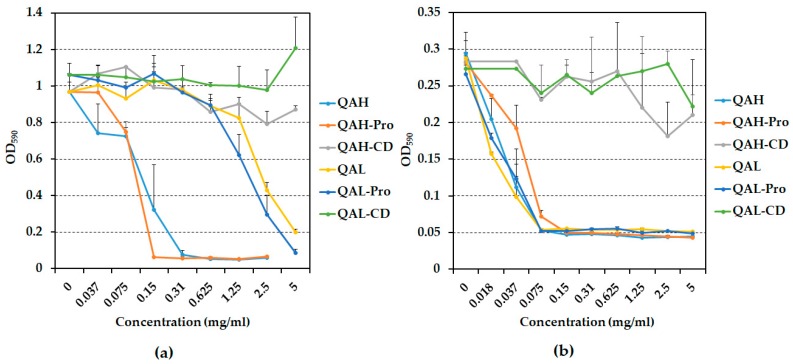
Absorbance values recorded after 20 h of incubation of bacterial cells with different concentrations of various Ch-derivatives. Exponentially growing *P. aeruginosa* (**a**) or *S. epidermidis* (**b**) were incubated in Mueller Hinton broth (MHB) at 37 °C in static conditions for 20 h prior of measuring optical density at 590 nm. Graphs show mean values ± SEM from three independent experiments.

The antibacterial activity of different Ch-derivatives was tested against exponentially growing *P. aeruginosa* and *S. epidermidis* in terms of minimal inhibitory concentration (MIC) values and by measuring the optical density of bacterial suspensions exposed to different concentrations of the compounds for 24 h. QAH and QAH-Pro caused a dose-dependent reduction of the OD_590_ of *P. aeruginosa* with a complete inhibition of visible bacterial growth (MIC value) at the concentration of 0.31 and 0.15 mg/mL, respectively (Table 2, Figure 1A). Against the same bacterial species, the low MW Ch-derivatives (QAL and QAL-Pro) were less active in inhibiting bacterial growth than QAH and QAH-Pro, showing MIC values of 5 mg/mL (Table 2, Figure 1A). Finally, QAH-CD and QAL-CD were completely inactive in reducing OD_590_ of *P. aeruginosa* up-to the concentration of 5 mg/mL (Table 2, Figure 1A). Regarding *S. epidermidis*, both high (QAH and QAH-Pro) and low (QAL and QAL-Pro) MW Ch-derivatives caused a dose-dependent reduction of OD_590_ with MIC values of 0.075 mg/mL for all four compounds (Table 2, Figure 1B). Again, QAH-CD and QAL-CD showed no effect on bacterial growth until the concentration of 5 mg/mL.

**Figure 2:**
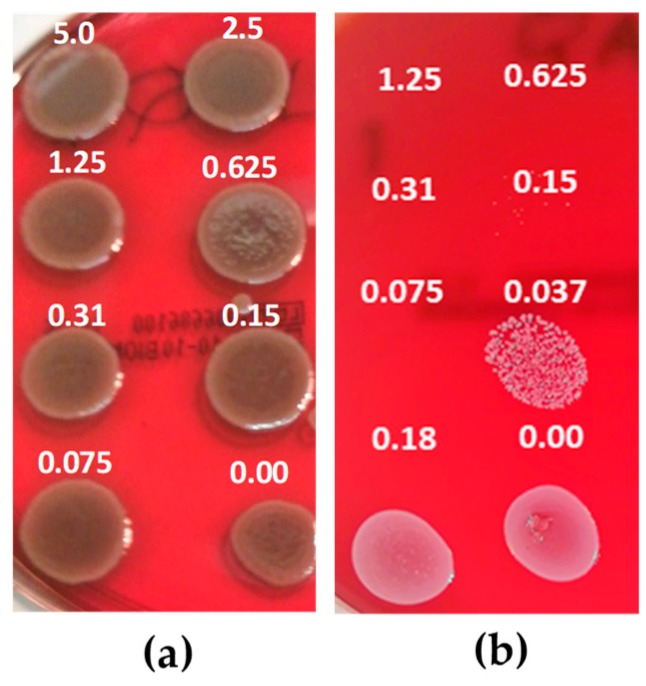
MBC determination of Ch-derivatives against *S. epidermidis* and *P. aeruginosa*. Exponentially growing cultures of *P. aeruginosa* (**a**) and *S. epidermidis* (**b**) were exposed to different concentrations of Ch-derivatives (white numbers; mg/mL) for 24 h. An aliquot of 10 µL from each well was then spot-plated on the surface of agar blood plates and incubated for overnight at 37 °C. MBC was determined as the lower concentration of each compound resulting in the growth of 5 colonies or less per spot. Results obtained in a representative experiment for QAL are shown.

Next, ability of QAH, QAH-Pro, QAL and QAL-Pro to exert a bactericidal effect was evaluated, determining the minimal bactericidal concentration (MBC) values against exponentially growing *P. aeruginosa* and *S. epidermidis*. As shown in Table 2 and in Figure 2 for a representative experiment, none of the compounds tested exhibited a bactericidal effect (reduction of at least 3 Log unit in the CFU number) against *P. aeruginosa* up-to the concentration of 5 mg/mL. In contrast, a striking bactericidal effect was observed against *S. epidermidis* with MBC values ranging from 0.075 to 0.31 mg/mL depending on the compound tested (Table 2 and Figure 2).

### 2.2. Ability of Quaternized Ch-Derivatives to Prevent Biofilm Formation by P. aeruginosa and S. epidermidis

**Figure 3:**
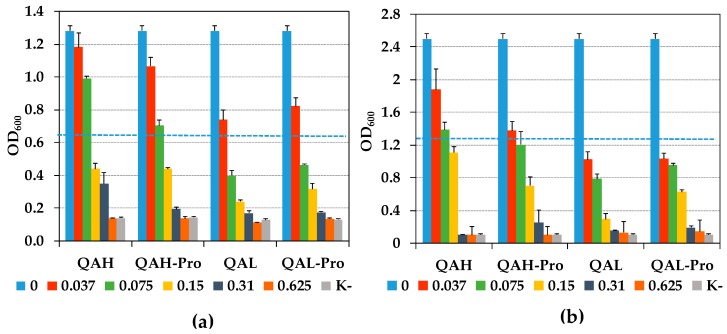
Inhibition of *P. aeruginosa* ATCC 27853 (**a**) and *S. epidermidis* ATCC 35984 (**b**) biofilm formation by different chitosan derivatives. Overnight cultures of each bacterial species were diluted 1:100 and incubated with different concentrations of four chitosan derivatives. After 18 h of incubation at 37 °C, biofilm formation was quantified by crystal violet staining. The Figure depicts the mean values ± SEM of at least two independent experiments performed in duplicate. Dotted lines indicate a 50% reduction in biofilm formation. Concentrations are in mg/mL. K-: wells without bacteria.

The ability of QAH, QAH-Pro, QAL, and QAL-Pro to inhibit the biofilm formation of *P. aeruginosa* and *S. epidermidis* was tested by a standard micro-well plate assay. Quantification of total biofilm biomass in the presence of different concentrations of each compound was evaluated by staining with crystal violet (CV), a dye able to stain both bacterial cells and the extracellular matrix. As shown in Figure 3, a dose dependent ability of all four the compounds to inhibit biofilm formation of both *P. aeruginosa* and *S. epidermidis* was observed as compared to cells incubated in medium only. In particular, a 50% reduction in biofilm formation was obtained at concentrations ranging from 0.037 to 0.15 mg/mL depending on the compound and bacterial species tested. The concentration at which an antimicrobial compound exerts antibiofilm activity is considered indicative of its antibiofilm mode of action [22]. Interestingly, as shown in Supplementary Figure S1 for a representative experiment, in the case of *P. aeruginosa* a reduction of 50% in biofilm formation was obtained at concentrations unable to inhibit bacterial growth. This observation suggests that the Ch-derivatives could prevent biofilm formation by a biofilm specific mechanism (e.g., interference with adhesion or with the bacterial communication machinery that promote biofilm production). In contrast, for 3 out of 4 compounds tested, 50% inhibition of *S. epidermidis* biofilms was obtained at concentrations able to inhibit bacterial growth (Figure S1) indicating that, in this case, prevention of biofilm might be very well due to a classical inhibitory/bactericidal effect of the compounds against planktonic biofilm forming cells.

### 2.3. Functionalization of Titanium Oxide Surfaces

**Figure 4:**
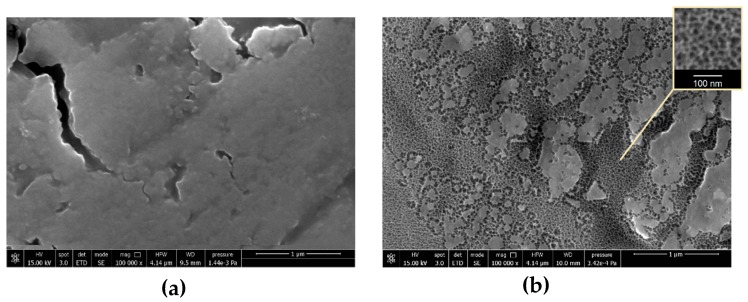
FE-SEM (100,000×) micrographs of titanium samples. (**a**) Untreated titanium (Ti) and (**b**) titanium subjected to etching, Ti-Ox, with inset showing nanopits.

Plain titanium and alloys thereof are the most commonly used materials for orthopedic prosthesis implants. Major factors for implant success are osteo-integration and prevention of microbial colonization. In this context, modification of the prosthesis interfaces can favor the implant success without altering the physical characteristics of the whole device [23]. Concerning titanium surface functionalization, five consecutive steps were applied to covalently bind the polymers under study to the surface of 7 × 7 mm^2^ titanium samples (Supplementary materials Scheme S1). The different stages of the protocol were worked out and the resulting characteristics of surfaces were evaluated by microscopic, colorimetric and spectroscopic techniques. Each stage leading to polymer binding to the titanium oxide surface was monitored by attenuated total reflection/Fourier transform infrared spectroscopy (ATR/FT-IR). The results of the analyses are shown in Figures S2–S6 under Supplementary Materials. With respect to untreated titanium surface (Ti), 10 min of etching treatment (Ti-OX) determined a widespread nanotexture, as visualized in high-resolution SEM micrographs (100,000× magnification) with nanopits of 19.5 ± 4.5 nm average diameter, at the surface opening (Figure 4).

Surface silanization is frequently adopted to stably anchor new chemical features [24], and a preliminary screening of the condition described in the literature was performed, mainly concerning the use of different solvents such as ethanol, water or anhydrous toluene [25,26,27] (Supplementary material for details). The choice was then dictated by the quantification of amino moieties density on APTES silanized surfaces as obtained by using the picric acid spectrophotometric method [28]. The primary amino groups of the superficial film react with picric acid to form the colored picrate group that is released from surface to solution. The amino group density is calculated from the characteristic absorbance of picrate. Since the method involves reaction and elution steps separated by intermediate rinsing of unreacted picric acid, the bound picrate elution does not expose unreacted amino groups to further reaction. APTES amino density on treated titanium surfaces resulted most effective under toluene conditions, 59.2 × 10^−2^ ± 3 ×∙10^−2^ nmol/mm^2^. As for the subsequent immobilization of Ch- derivatives, QAH, QAL and QAH-Pro polymers were grafted on titanium surface, named Ti-QAH, Ti-QAL, and Ti-QAH-Pro respectively. The covalent grafting was performed by using glutaraldehyde as crosslinker, either as single layer grafting or, concerning QAH-Pro, as multi-layering conjugation (Ti-QAH-Pro_ML; supporting materials). Additionally, also the use of hexamethylene diisocyanate (HDMI) as alternative crosslinker was preliminary evaluated for both QAH and QAL polymers (named Ti-QAH-Cyan and Ti-QAL-Cyan, details in Supplementary materials). The amount of bound material was quantified by size exclusion chromatography (SEC) and, as displayed in Table 3, QAH was the most effectively grafted polymer, both by glutaraldehyde and HDMI linking, resulting in 3.9 µg/mm^2^ and 4.5 µg/mm^2^, respectively. Differently, QAL was linked to a lower extent, 0.7 µg/mm^2^ and 1.0 µg/mm^2^, for Ti-QAL and Ti-QAL-Cyan respectively. QAH-Pro grafting was merely appreciable by multi-layering only.

QAH grafting resulted the most reproducible (Ti-QAH) and was selected for further characterizations in terms of grafting homogeneity and antiadhesion capability.

### 2.4. Surface Features of Polymer Grafted Titanium Surface

Ti-QAH and Ti-Ox underwent further characterizations in order to assess surface morphology and chemistry. Both Ti-QAH (20.45 ± 0.636 mV) and Ti-Ox (14.3 ± 0.458 mV) presented positive charges at pH 3.8, with significantly higher values for the polymer grafted ones. A similar trend was observed also in diluted PBS (<10 mM), at analogous pH to that of MHB bacterial medium (pH 7.3), where Ti-QAH resulted in (−28.1 ± 4.5 mV) and Ti-Ox in (−40.5 ± 3.5 mV), thus confirming a screening of titanium surface, resulting in lower net charge.

**Figure 5:**
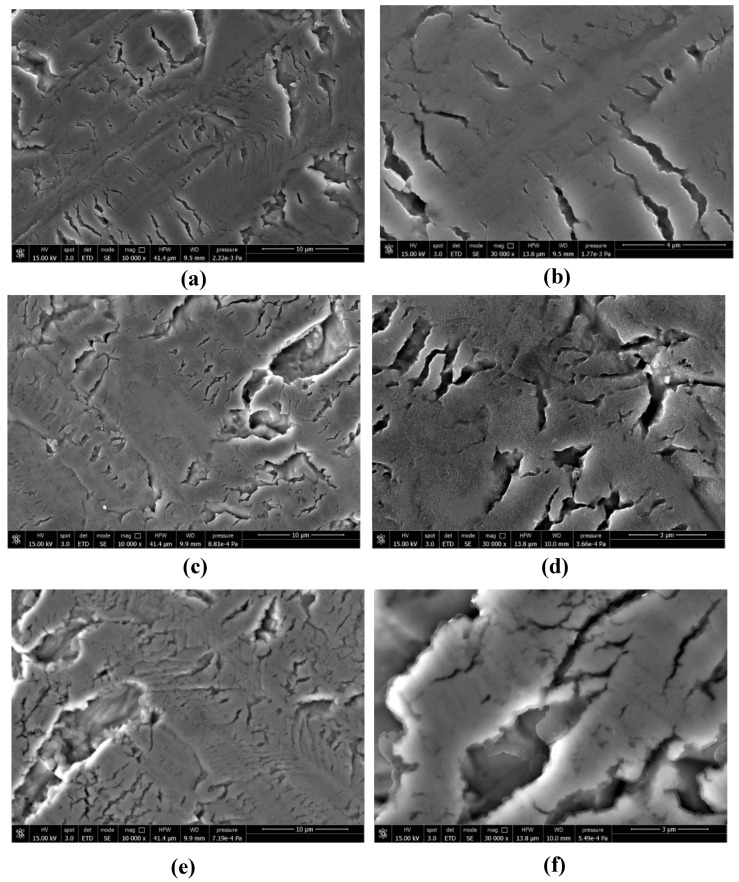
FE-SEM (10,000× and 30,000×) images of titanium samples. (**a**) and (**b**) titanium as such, Ti; (**c**) and (**d**) titanium subjected to etching, Ti-Ox; (**e**) and (**f**) titanium with grafted QAH, Ti-QAH.

The SEM images of titanium samples surfaces as such, after etching and after grafting are shown in Figure 5. The untreated titanium surface, only subjected to washing to remove possible organic materials, presented typical flat titanium lamination and fracture with sharp edges; after etching treatment the surface was still displaying the fractures, but surface nano-roughness was clearly evident. After polymer grafting (Ti-QAH), the surface showed a “muffled” appearance, due to the presence of organic material, which was even more evident in the 30,000× magnification micrograph. Here, a very thin coating was masking the underneath titanium cracks.

EDS measurements (Table 4) confirmed the occurred oxidation, silanization, and grafting on titanium surface. Titanium was the most abundant element detected for all specimens. However, its percentage diminished from Ti to Ti-Ox, balanced by an increase of oxygen value. Concerning the polymer grafted samples, silicium, carbon, and nitrogen were also detected to proof the occurred silanization and polymer grafting.

**Figure 6:**
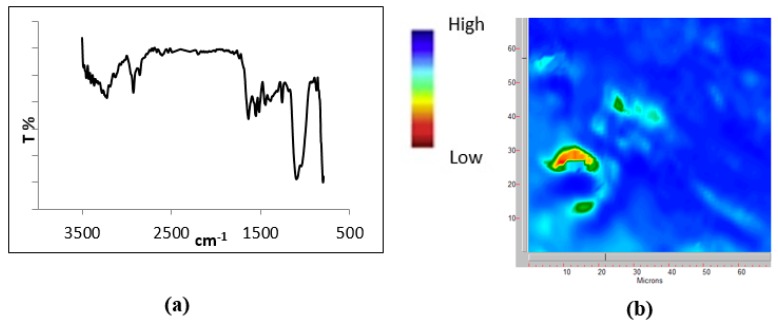
Micro-ATR of Ti-QAH sample. (**a**) ATR/FT-IR of the QAH polymer grafted to the titanium surface. (**b**) Distribution map of the matrix based on the band at 1639 cm^−1^.

The homogeneous distribution of QAH on Ti-QAH specimens was evaluated by FTIR spectroscopic imaging (Figure 6). Firstly, the ATR spectrum of Ti-QAH (Figure 6a) displayed the QAH characteristic bands with the overlapping of the stretching band of the imine function C = N at 1639 cm^−1^. The spectrum evidenced also the presence of polymer characteristic peaks, such as: 1657 cm^−1^ (v C=O), 1590–1564 cm^−1^ (residual –NH_2_ bending and 2^nd^ amide band), 1480–1456 cm^−1^ (CH_2_ and CH_3_ bending), 1392 cm^−1^ (CH_3_ bending), 1375 cm^−1^ (CH_2_ bending), 1320 cm^−1^ (ν C-N of N-acetylglucosamine), 1103 cm^−1^ (ν C-N of tertiary amine), 1025 cm^−1^ (ν C-O-C). The obtained FT/IR surface map (Figure 6b) displayed a homogeneous distribution of the polymer on the Ti-QAH surface, except for random narrow regions, due to the grooves as already observed on SEM micrographs.

### 2.5. Ability of Ti-QAH to Prevent Adhesion of S. epidermidis

**Figure 7:**
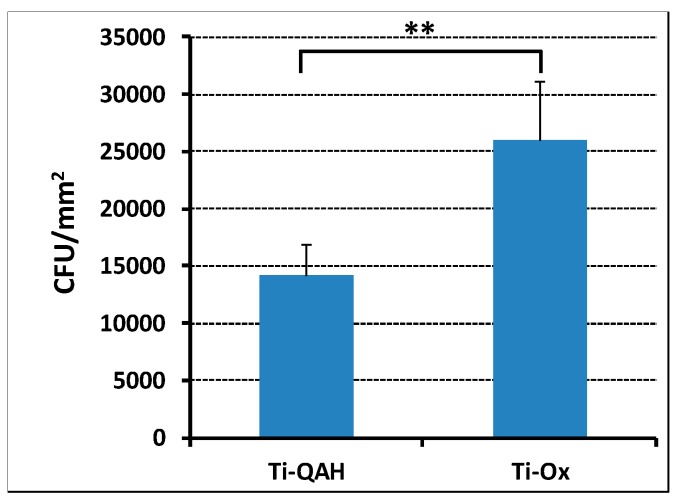
Ability of titanium functionalized with QAH to inhibit adhesion of *S. epidermidis.* Titanium plates (7 × 7 mm) functionalized with QAH (Ti-QAH) or just etched (Ti-Ox) were incubated with *S. epidermidis* for 6 h. After gentle washing and sonication to detach adhered bacteria, suspensions were serially diluted and plated for CFU count. ** *p* < 0.01, Student’s t-test for paired samples.

Due to its marked ability to colonize indwelling medical devices, *S. epidermidis* is considered one of the major bacterial species associated with implant-related infections [29]. For this reason, we investigated the ability of QAH functionalized titanium surfaces (Ti-QAH) to prevent adhesion of this bacterial species. To this aim titanium plates either covalently grafted with QAH or just etched were incubated statically for 6 h with 1 × 10^6^ CFU/mL of *S. epidermidis*. After gentle washing to remove non-adhered bacteria and mild sonication to detach bacteria from titanium plates, the number of CFU was determined and expressed as CFU/mm^2^. As shown in Figure 7, functionalization was able to reduce the number of adhered bacteria of approximately 45% as compared to control plates with a statistically significant difference in the CFU/mm^2^ between the test sample and the control.

## 3. Discussion

Over the last few years quaternized Ch-derivatives have attracted considerable interest as alternative to Ch as antimicrobial agents [30]. Indeed, the increment in water solubility at physiological pH and the improved antimicrobial activity provided by the quaternary modifications enlarge the potential biomedical applications of Ch in the field of anti-infective interventions. A number of quaternized Ch-derivatives have been synthetized following different methods, and their antimicrobial properties tested against different microbial species [30,31,32]. Nonetheless, as outlined in the following paragraph, discrepancies have been reported among different studies on the antibacterial activity of Ch and its quaternary derivatives [10,30,33,34] warranting further investigations to explore fully the potentials of such molecules as antimicrobials. Discrepancies on the spectrum of activity, antimicrobial potency, effect of pH or substitutions might be due to several reasons related to Ch itself (e.g., type of substitutions, MW, deacetylation degree, viscosity, solvent and concentrations used, solubility etc.) or to the environmental conditions (e.g., test strains and their physiological state, medium used for the antimicrobial susceptibility assay, ionic strength, pH etc.). In our study, a fine description and characterization of the Ch-derivative tested as well as of the experimental conditions adopted was provided, allowing a reliable evaluation of Ch-derivative activity at physiological pH, not only against planktonic cells but also against microbial biofilms.

### 3.1. Antibacterial Activity Toward P. aeruginosa and S. epidermidis

In the present study, we evaluated the antimicrobial and antibiofilm activity of different quaternized Ch-derivatives that have been previously characterized for their pharmacological properties demonstrating significant muco-adhesive properties and ability to promote wound healing [13,14,15]. The various Ch-derivatives tested showed differential abilities to inhibit the growth of two medically relevant bacterial species, *P. aeruginosa* and *S. epidermidis*, representative of Gram-negative and Gram-positive bacteria respectively. Overall, *S. epidermidis* displayed higher susceptibility to both high and low MW Ch-derivatives than *P. aeruginosa*. This finding is in agreement with a previous study reporting a stronger activity of quaternized N-aryl Ch-derivatives against the Gram-positive *Staphylococcus aureus* than the Gram-negative *Escherichia coli* [33]. Another report found that N-2-hydroxypropyltrimethylammonium (HACC) derivatives, with a high degree of substitution (88%), had no activity against Gram-negative bacteria such as *E. coli* and *P. aeruginosa*, but were active towards the fungus *C. albicans* and the Gram-positive *S. aureus*, *S. epidermidis* and *Bacillus subtilis* [34]. Overall, these findings suggest that the high lipophilic outer membrane of Gram-negative bacteria may function as an efficient barrier against hydrophilic macromolecules such as Ch-derivatives. Combining Ch-derivatives with agents (such as EDTA) able to destabilize the outer membrane of Gram-negative bacteria as well as to sequester positively charged medium components could help in increasing their activity against this group of bacteria [35]. However, a contrasting study, aiming at correlating the antibacterial activity of Ch and the surface characteristic of bacterial cell wall, found that the negative charge on the cell surface of Gram-negative bacteria (*P. aeruginosa*, *Salmonella typhimurium* and *E. coli*) was higher than that of Gram-positive one (*Staphylococcus aureus* and *Streptococcus faecalis*) resulting in more Ch adsorbed and higher inhibitory effect against the Gram-negative bacteria [36]. The exact mechanisms of the antibacterial action of quaternized Ch-derivatives have to be fully elucidated yet, but it seems that their antimicrobial activity mostly depends on the degree of substitution of the grafting groups that, in turn, affect the positive charge density [30,31,32]. It is reported that the polycationic nature of the molecule is a prerequisite for its antibacterial activity as it favors the electrostatic interaction with the negatively charged bacterial surface. This in turn, causes extensive cell surface alteration, leakage of intracellular components and ultimately results in cell death. Other intrinsic factors that may influence the antimicrobial activity of Ch-derivatives are their structure, conformation and MW [30]. In the present study, high and low MW derivatives were equally active against *S. epidermidis* in terms of MICs and MBCs, while high MW derivatives exhibited stronger activity against *P. aeruginosa* than low MW ones. Interestingly, protection of thiol groups in QAL-Pro and QAH-Pro did not exert a major effect on MIC values, while Ch-derivatives grafted with methyl-β-cyclodextrin (QAH-CD and QAL-CD) completely lost their inhibitory capacity in the experimental conditions and at the concentrations tested. Thus, it is possible that the right balance between steric hindrance, charge distribution and properties of the targeted bacterial cell surface is needed for the optimal inhibition of microorganisms by Ch and its derivatives.

### 3.2. Prevention of Biofilm Formation

It is now widely recognized that the biofilm mode of growth is the predominant mode of growth of bacteria both in the environment and in the host and that biofilm cells differ substantially as compared to their planktonic counterpart in terms of growth rate, gene expression and structural properties [3]. Thus, testing the antimicrobial properties of new compounds not only against exponentially growing bacterial cells but also against biofilms is mandatory. Ability of quaternized Ch-derivatives to interfere with biofilm formation is a promising but still relatively poorly investigated subject [37,38]. In the present study, the tissue culture plate method was used to test the ability of QAH, QAH-Pro, QAL and QAL-Pro to prevent biofilm formation by *P. aeruginosa* and *S. epidermidis*. Such widely used method allows quantifying the total biofilm biomass that comprises both bacterial cells and the extracellular polymeric substance they produce. The Ch-derivatives tested showed a striking ability to inhibit biofilm formation by both *P. aeruginosa* and *S. epidermidis*. Overall, low MW derivatives (QAL and QAL-Pro) inhibited biofilm formation at concentrations slightly lower than the high MW ones (QAH and QAH-Pro) suggesting that the latter may less efficiently diffuse into the structure of the forming biofilms and target bacterial cells. Of note, as reported above, QAH and QAH-Pro were more active against planktonic cells than QAL and QAL-Pro (at least against *P. aeruginosa*), underlying that the antibacterial and antibiofilm activities of a molecule are not always coincidental. Interestingly, in the case of *P. aeruginosa*, the antibiofilm effect was observed at concentrations unable to inhibit bacterial growth in the conditions adopted to promote biofilm formation (stationary phase cells of *P. aeruginosa*, incubated in 10% TSB for 24 h). This finding suggests that the formation of biofilm was not prevented by a “classical” antibacterial effect, but possibly by interfering with specific features of the biofilm mode of growth [21]. In many microorganisms, including *P. aeruginosa*, biofilm formation is (co-)regulated by Quorum Sensing (QS), the bacterial communication machinery that allows coordinated gene expression in response to cell density and local conditions [39]. Thus, it is tempting to speculate that the Ch-derivatives tested herein could somehow interfere with QS signals that promote biofilm formation and expression of virulence factors. This hypothesis is supported by recent reports that describe ability of extracted Ch to down regulate the expression of QS regulated genes in *P. aeruginosa* [40,41].

As regards *S. epidermidis*, the prevention of biofilm formation mostly occurred at concentrations able to inhibit growth in the adopted conditions suggesting that the inhibitory mechanism may at least in part relies on a classical antibacterial effect against planktonic biofilm forming bacteria. One of the main factor promoting the accumulation of biofilm cells in *Staphylococcus* is the production of the extracellular polysaccharide intercellular adhesin (PIA), a polymer of β-1,6-linked N-acetyl-glucosamine with partially N-deacetylated amine groups, synthesized and secreted by the gene products of the *ica* operon, *ica*A, *ica*D, *ica*B and *ica*C [42]. Interestingly, ability of hydroxypropyltrimethyl ammonium chloride Ch to inhibit *ica*A transcription has been previously described [37] suggesting the possibility that quaternized Ch-derivatives may also act by interfering with s specific processes of the biofilm growth mode.

### 3.3. Titanium Functionalization and Prevention of Bacteria Adhesion

Nowadays medical interventions are heavily dependent on the use of biomaterial implants and medical devices (e.g., orthopedic implant and prosthetic joints). The introduction in clinical practice of these devices has allowed not only to ensure a better quality of life for many patients but also to save patients’ life in critical circumstances. Nevertheless, there is a major drawback in the use of medical devices that is the possibility they can be colonized by bacterial cells that subsequently may promote biofilm development. Nowadays, biomaterial-associated infections are a major concern in modern medicine accounting for more than half of healthcare-associated infection and greatly contributing to morbidity, mortality and sanitary costs. Bacteria can reach a biomaterial in several ways and at different times post-implantation. Most commonly, colonization arises during insertion of the implant by bacteria present in the operating room or normally populating the surgeons’ skin. After the initial adhesion phase, bacterial cells start to proliferate and produce a complex extracellular matrix that surround and protect them and promote their stable colonization of the biomaterial. Once the biofilm is formed it is very difficult to eradicate as in this form bacterial cells are extremely tolerant to antibiotics and clearance from host immune system. Therefore, it is recognized that the best way to prevent this process is to interfere with the early adhesion phase via the development of new materials that resist or prevent bacterial adhesion and subsequent biofilm formation [28]. In this context, in the present study we aimed to functionalize titanium plates with the Ch-derivatives under investigation to evaluate whether such strategy could reduce adhesion of *S. epidermidis*, one of the major bacterial species involved in implant colonization [28].

Titanium-based biomaterials are currently the best and most widely used materials in the manufacture of orthopedic and dental implants, because of their high strength, low weight, excellent corrosion resistance and good biocompatibility. For instance, the clinical long-term success rate of titanium-based dental implants is presently 87.8%, in a follow-up period of 36 years [43]. Thus, titanium and alloys-based devices are continuously investigated and upgraded for their application and commercial exploitation in the biomedical field. Surface treatments, leaving unaltered the chemical-physical features of optimized devices, is presently the most deeply investigated aspect. Despite the extensively described correlation between micro/nano-scale features and osseointegration, the implant failure induced by microbial adhesion and biofilm accumulation is still an open question [44]. It is proven that titanium surfaces do not prevent bacterial adhesion by themselves [45] and their coating, either physical or covalent, with antimicrobial actives is a thriving and challenging field of research [28].

The anchoring of Ch to titanium surfaces has recently demonstrated the possibility of improving the susceptibility to antimicrobial treatments and reducing the adhesion of bacteria to the prosthetic device [46]. Since the chemical/physical surface treatments of commercial devices, providing micro/nano-textures, are improving host cell adhesion for better osseointegration but also bacterial colonization [47], it appears mandatory to assess the bacterial anti-adhesion functionality in comparison to treated titanium surfaces displaying micro/nano-texture.

In the present study, three Ch-derivatives were selected among those screened as bactericidal and inhibiting agents for biofilm formation. Their covalent grafting on chemically treated (etched) titanium surfaces was performed and quantified in terms of polymer surface density (µg/mm^2^). The chemical piranha treatment was performed for a short time, to achieve a surface modification, and not an interconnected porosity, which would cause a significant alteration of the mechanical properties of material, and a mass loss by corrosion [48]. Compared to other titanium surface passivation methods, such as the treatment with HNO_3_, the use of the piranha solution generally causes an increase of the oxide layer thickness and a nano-scaled roughness rugosity [49], which were confirmed by EDS analysis and SEM microscopy. The subsequent linking step, involving APTES and glutaraldehyde, has been widely exploited for the immobilization of peptides or enzymes in the microarray technology [50] and can be adapted for the immobilization of polymers bearing amino moieties, such as Ch [51]. Surface silanization was quantified by picric acid assay and confirmed by EDS elemental analysis. Concerning the linker, glutaraldehyde is a five-atom arm spacer, promptly reactive to amine moieties. Among tested polymers, a trend was observed for the grafting effectiveness, namely QAH > QAL > QAH-Pro. A similar trend was also noticed when a longer spacer arm (10-atoms, HMDI) was used. Being irrespective of the adopted spacer, it is feasible that polymer MW has an effect in the grafting yield, since that is the main structural difference between QAH and QAL, having similar quaternarization substitution, instead. Concerning QAH-Pro, it was possible to record an appreciable grafting only with the multi-layering approach, similarly to what reported by Peng et al., 2015 [52] for HACC Ch-derivative. Considering that these polymers are highly substituted (>80% of quaternarization) and acetylated in the 5–10% range, the reactive amino moieties on polymer backbones, available for covalent grafting, are relatively few. Additionally, the steric hindrance and the charge repulsion of the pendant quaternary ammonium side chains are considered to play a role in the observed low grafting results. Finally, the reactivity of the QAH-Pro macromolecules with the activated support could have been reduced further, due to the presence of additional thiol functionalization and protection.

The grafting of QAH resulted homogeneously distributed on Ti surfaces, as confirmed by FT-IR and SEM imaging, additionally, it determined a statistically significant alteration of the net charge associated to passivated titanium surfaces. Concerning the antiadhesion effect of the grafted polymer, it was evaluated by quantifying the number of viable bacterial cells adhered on the surface and the results obtained are in line with what reported by Peng et al. 2015, for HACC quaternized Ch [51]. In this latter case, however, a Ch bearing a lower percentage of quaternarization (18%) was used, without expressing the density of polymer grafting, and performing the comparison of bacterial adhesion toward an untreated Ti surface. Beyond the extensive antimicrobial characterization of the Ch-derivatives object of the present paper, we focus our attention also on the quantification of the polymer surface density, considering that this aspect is a central parameter, to be correlated to the antiadhesion property, useful also for future additional experiments and exploitation.

## 4. Materials and Methods

### 4.1. Polymeric Materials

Giusto Faravelli FG90 (CSH) and its lower MW derivative, obtained by oxidative depolymerization with NaNO_2_ (CSL) were used as precursors for the preparation of quaternized chitosans as described in previous studies [53]. The relevant high and low MW quaternary ammonium-Ch conjugates (QAH and QAL, respectively) were prepared as described by Zambito, et al., 2006 [54]. High and low MW multifunctional quaternary ammonium-Ch conjugates substituted with protected thiol groups (QAH-Pro and QAL-Pro, respectively) were obtained as previously described [55]. The preparation of multifunctional quaternary ammonium-Ch derivatives of high or low MW, conjugated with methyl- β-cyclodextrin (QAH-CD or QAL-CD) followed a reported protocol [19,20]. The main features of the polymers assayed in the study are reported in Table 1, details of the characterization techniques, including spectroscopy analysis, are reported in Supplementary Figures S7–S10.

### 4.2. Bacterials Strains

The reference strains *Staphylococcus epidermidis* ATCC 35984 and *Pseudomonas aeruginosa* ATCC 27853 were used for the study. For the preparation of stock cultures, bacterial strains were grown in Muller Hinton broth (MHB) or Tryptone Soy Broth (TSB) (Oxoid, Basingstoke, Hampshire RG24 8PW, UK) until mid-log phase, subdivided in aliquots and kept frozen at −80 °C until use. For colony forming unit (CFU) count, serially diluted bacterial suspensions were plated in duplicate on Tryptone Soy agar (TSA)(Oxoid) and incubated at 37 °C for 24 h.

### 4.3. Determination of Minimal Inhibitory Concentrations (MICs) and Minimal Bactericidal Concentrations (MBCs)

The susceptibility of *P. aeruginosa* and *S. epidermidis* to high and low MW quaternized Ch-derivatives was assessed in terms of MICs values according to the standard microdilution method (Clinical and Laboratory Standards Institute–CLSI, 2018). Briefly, bacteria were grown in Mueller-Hinton Broth (MHB, Oxoid), until exponential growth phase and diluted in the same medium to reach a final density of 5 × 10^6^ CFU/mL. A volume of 10 μL of the bacterial suspensions was added to 90 μL of MHB in a 96-well plate in the absence (viability control) or in the presence of the compounds at different concentrations. MIC values were defined as the lowest concentration of each compound resulting in the complete inhibition of visible growth after 24 h of incubation at 37 °C. The effects of different concentrations of each compound on bacterial growth were assessed by measuring the OD_590_ of each well in an automatic microplate reader (Bio-Rad Laboratories, Segrate, Italy). For determination of MBCs, 10 µl from each well were spot-plated on the surface of 9 cm diameter blood agar plates. Plates were incubated over-night at 37 °C and the MBC determined as the lower concentration of each compound resulting in the growth of five colonies or less per spot.

### 4.4. Biofilm Inhibition Assays

Ability of the compounds (QAH, QAH-Pro, QAL, QAL-Pro) to inhibit biofilm formation of *S. epidermidis* and *P. aeruginosa* was assessed by CV staining. To this aim, the two bacterial strains were grown in agitation overnight in MHB (*S. epidermidis*) or TSB (*P. aeruginosa*). Following incubation, bacterial suspensions were diluted 1:100 in MHB (*S. epidermidis*) or in 10% TSB (*P. aeruginosa*) and seeded into wells of flat-bottom polystyrene 96-well microtiter plates (Corning Costar, Lowell, MA, USA) in the presence of different concentrations of each compound (final volume 100 µL). Wells containing bacteria and no compounds or medium without bacteria were prepared as positive and negative controls, respectively. Plates were incubated over-night in static conditions at 37 °C to let biofilms grow. Following incubation, wells were gently washed with distilled water for three times to remove non biofilm-embedded bacteria and plates were allowed to dry at 60 °C for 1 h. To stain biofilms, 150 µL of a solution of 0.5% CV was added to each well and plates were incubated at room temperature for 15 min. Wells were repetitively washed with distilled water until no blue color was coming out from the wells. Plates were air-dried at 37 °C for 30 min and 200 µL of 33% acetic acid added to each well to extract biofilm-bound CV. After a further incubation at room temperature for 15 min, OD_570_ was measured on a microplate reader.

### 4.5. Functionalization of Titanium Surface

The modification of titanium surfaces by QAH, QAL, or QAH-Pro grafting was performed by modifying the protocol of Martin et al. 2007 [27] according to the following stages:

1. Washing: titanium samples, each of 0.25 mm thickness, two faces of 7 × 7 mm^2^ were cut from a commercial titanium foil (Titanium foil, thickness 0.25 mm, Sigma Aldrich). The samples were cleaned by sonication at 40 MHz (Sonica Ultrasonic Cleanermod 2200 ETH, SOLTEC) in acetone, ethanol and distilled water, in sequence, 10 min at 25 °C and dried with filter paper by capillarity.
2. Etching: Each sample was treated with 3 mL of a 2:1 30% H_2_SO4-H_2_O_2_ solution (piranha solution), vortexed 10 s and left to react in a shaker water bath 10 min, 80 rpm. Finally, the samples were washed 3 times with 20 mL of distilled water and wiped dry with filter paper by capillarity, subsequently the drying was completed under vacuum at 37 °C.
3. Silanizing and curing: the deposition of 3-aminopropyltrietoxysilane (APTES, Sigma-Aldrich) was achieved by immersing each titanium sample in 5 mL of a 5% (*v*/*v*) APTES solution in toluene under inert N_2_ atmosphere and leaving the reaction proceeding 24 h in a shaker water bath (25 °C, 80 rpm). Next, the samples were washed with 20 mL each of toluene, ethanol and deionized water, dried by wiping with filter paper, then under vacuum, followed by heating to 110 °C in an oil bath for 20 min under an N_2_ stream (curing). Additional details are reported in the supporting info file.
4. Spacer insertion: glutaraldehyde was used as the spacer between APTES and each polymer. For spacer insertion each sample was immersed in 5 mL of a 5% (*v*/*v*) glutaraldehyde solution in deionized water and the reaction was left to proceed 24 h in a shaker water bath (25 °C, 80 rpm). Thereafter, the samples were washed three times by dipping in 20 mL of deionized water in sequence and wiped dry by capillarity with filter paper. Additional details are reported in the supporting info file.
5. Grafting of Ch-derivatives: the reaction between the aldehyde functional groups and the polymer derivatives was carried out by dipping each sample in 2 mL of a 0.5 mg/mL polymer solution and leaving the reaction proceed in a shaker water bath 24 h at 25 °C and 80 rpm. Then the samples were washed again with deionized water and vacuum dried. Additional details are reported in the supporting info file.

All samples were kept under vacuum until their use or instrumental characterization.

### 4.6. Characterization of Polymer Grafted Titanium Surfaces

#### 4.6.1. Quantification of APTES Amino Moieties with Picric Acid

Each silanized sample to be analyzed was treated with 1 mL of a 5% (*v*/*v*) triethylamine (TEA) solution in methanol over 10 min, then washed 3 times with 1 mL methanol. Subsequently each sample was treated with 1 mL of 1% aqueous picric acid for 10 min, then 5-fold washed with 1 mL of methanol for 2 min each. The picrate was eluted 10 min with 1 mL of a 5% (*v*/*v*) TEA solution in methanol and the resulting solution was spectrophotometrically analyzed (Shimadzu UV-2101 PC). The absorbance was measured at 390 nm and normalized on 445 nm (LOD 0.293 µM and LOQ 0.995 µM).

#### 4.6.2. Quantification of Polymer Grafting by Size Exclusion Chromatography (SEC)

The apparatus was Shimadzu LC-20 AT, with injection valve 20 μL Rheodyne, refraction index detector LC-30 PerkinElmer, column furnace HPLC10A Athena Technology, column Jordi Resolve xStream GPC 7.8 mm Mixed Bed 30 cm, Jordi Labs, mobile phase 3% aqueous acetic acid, flow rate 1 mL/min at 40 °C. Calibration curves were built for high MW Ch (QAH), low MW Ch (QAL) and QAH-Pro, with the following limit of detection (LOD) and limit of quantification (LOQ): QAH LOD 0.06 mg/mL & LOQ 0.20 mg/mL; QAL LOD 0.04 mg/mL & LOQ 0.13 mg/mL; QAH-Pro LOD 0.06 mg/mL & LOQ 0.21 mg/mL. The quantification of polymers grafted on titanium surface was carried out indirectly, by comparing the concentration of the polymer solution before and after grafting. The polymer amount bound to the surface is expressed as μg/mm^2^.

#### 4.6.3. Surface Zeta Potential

Surface zeta potentials of etched titanium (Ti-ox) and QAH grafted titanium (Ti-QAH) samples were determined by electroosmotic flow mapping (EOFM) technique, by using a Zetasizer Nano ZS, equipped with the surface accessory, and the surface potential dip cell (ZEN1020). The specimens were cut into squares of side 3.5 mm, attached to the peek holder by using cyanoacrylate glue, and cleaned by sonication in deionized water for 5 min at 25 °C. The was measured according to the manifacturer instruction (Malvern, UK) by using Ch nanoparticles as tracer, prepared according to Piras et al. 2015 [56], re-dispersed in water and displaying the following features: diameter 252 ± 7.7 nm (PI 0.324); 23.3 ± 0.7 mV at pH3.8 (SI file for tracer preparation details). All the experiments were run at 25 °C, using tracer particle concentration of 0.05% wt.

#### 4.6.4. Surface Morphology and Elemental Analysis

Surface features of titanium plates, such as morphology, chemistry and uniformity of the grafting were inspected by Field Emission - Scanning Electron Microscopy (FE-SEM; FEI Quanta 450 ESEM FEG) equipped EDS detector and by FTIR spectroscopic imaging (Cary 620 microscope coupled to Cary 660 Spectrometer, Agilent Technologies) with focal plane array (FPA) detector, under contact ATR mode. In this latter case, spectra were collected at 4 cm^−1^ resolution, pixel size of 5.5 m, with 512 scans and 3500–800 cm^−1^ spectral width. Concerning etched titanium (Ti-OX) specimens, the average diameter of the nanopits was evaluated over 50 measurements taken from 6 random fields (Image J 1.50i; Wayne Rasband National Institute of Health, USA) of the high resolution 100,000× SEM micrographs.

#### 4.6.5. Sterilization of Titanium Samples

The titanium samples were sterilized by exposing each side, in a Petri dish, to a UV ray source in a laminar flow hood (VBH C2, STERIL) for 30 min. Subsequently they were treated with 0.22 µm filtered solution of EtOH/H_2_O (70%/30%), according to the following protocol: immersion 1 h in 10 mL, washing twice with 10 mL (30 min each washing), immersion 20 h in the EtOH/H_2_O (70%/30%). After removal of the alcoholic solution, the titanium samples were left to evaporate dry 2 h in an aseptic hood, and finally placed in autoclave sterilized (Fedegari Autoclavi SPA, 20 min, 121 °C), glass bottles, which were then ferrule closed.

### 4.7. Bacterial Adhesion Inhibition Assay on Titanium Plates Functionalized with QAH

A stationary-phase culture of *S. epidermidis* was diluted in physiological solution (PS, 0.9% NaCl), added with 1% TSB, to obtain a bacterial suspension of approximately 1 × 10^7^ CFU/mL. Functionalized titanium plates and control plates (7 × 7 cm^2^) were incubated with 1 mL of the suspension in a 24 well plate at 37 °C with agitation at 400 rpm for 6 h. Then, the specimens were removed with sterile forceps, placed in another well and gently washed for three times with sterile PS to remove loosely adherent bacteria. Specimens were finally placed in 1 mL of PS, subjected to mild sonication for 5 min in a 100 W ultrasonic bath operating at 50 Hz, followed by rapid vortex mixing (30 s) to dislodge bacteria adhered to substrate. Serial ten-fold dilutions were performed and plated on TSA to calculate the viable count. The number of CFU on each sample surface was expressed relative to the surface area of the sample (CFU/mm^2^).

## 5. Conclusions

Due to the rising number of infections untreatable with the current arsenal of antibiotics, there is an urgent need to develop novel strategies to deal with this phenomenon. Bacteria that grow as biofilm, which render them even more tolerant to conventional antimicrobial treatment, sustain many of these infections. In this study, we provided new evidence for the possible application of quaternized Ch-derivatives as antibacterial, antibiofilm and/or anti-adhesive agents. With the exception of the QAH-CD and QAL-CD conjugates all the Ch-derivatives inhibited planktonic growth and biofilm formation of two clinically relevant bacterial species. Regardless of their MW and the presence of protected thiol groups on their chains, such Ch-derivatives showed the same efficacy against *S. epidermidis*, while the high MW derivatives proved to be more active against *P. aeruginosa* than the low MW ones. Regarding the antibiofilm properties, the Ch-derivatives with lower MW were surprisingly active at lower concentrations. These data altogether suggest that it is appropriate to modulate the chemical structure of polymers and use the most effective for each specific need. These polymers could be used in the form of solid particles to be inserted in pressurized containers to make spray plasters, or in solution to make liquid plasters. QAH derivative resulted effective also as bacterial adhesion inhibitor when covalently immobilized on titanium plates suggesting that optimized quaternized Ch coating on titanium implants could represent a good strategy to decrease infection rates associated with the implantation of medical devices.

The performed research highlights how polymer features can be tuned to amplify the intrinsic antimicrobial efficacy of Ch. In this view, a future combination of these natural derived Ch-based polymers is thought to provide enhanced protection from microbial infections by placing together the anti-adhesion effect with the inhibition of biofilm formation and antibacterial activity.

## Figures and Tables

**Table 1:** Main chemical characteristics of precursors (CSH and CSL) and Ch derivatives.

Polymers	MW, kDa; A_2_	DD ^a^, %	QD ^b^, %; n	TD ^c^, %	DSD ^d^, %	PD ^e^, %	CDD ^f^, %
CSH	379; 0.0009	−	−		−	−	−
CSL	131; 0.0008	−	−	−	−	−	−
QAH	428; 0.0001	7	85; 2	−	−	−	−
QAL	163; 0.0013	3	80; 2	−	−	−	−
QAH-Pro	460; 0.0001	7	85; 2	1.1	17.0	3.1	−
QAL-Pro	161; 0.0007	3	80; 2	1.2	14.9	2.3	−
QAH-CD	2344; 0.0001	7	85; 2	−	−	−	16
QAL-CD	633; 0.0001	3	80; 2	−	−	−	10

^a^ deacetylation degree; ^b^ quaternization degree, ^c^ thiolation degree; ^d^ disulfide degree; ^e^ protection with 6-mercaptonicotinamide (6-MNA) degree; ^f^ degree of substitution with methyl-β-cyclodextrin.

**Table 2:** MIC and MBC values in mg/mL of Ch-derivatives against *P. aeruginosa* and *S. epidermidis.*

Chitosan Derivatives	MICs		MBCs	
	***P. aeruginosa***	***S. epidermidis***	***P. aeruginosa***	***S. epidermidis***
**QAH**	0.31	0.075	>2.5	0.075
**QAH-Pro**	0.15	0.075	>2.5	0.31
**QAH-CD**	>5	>5	−	−
**QAL**	5	0.075	>5	0.075
**QAL-Pro**	5	0.075	>5	0.15
**QAL-CD**	>5	>5	−	−

**Table 3:** Polymer amounts grafted onto titanium surfaces (mean ± SD).

Sample	Linker	Polymer, µg/mm^2^
Ti-QAH	Glutaraldehyde	3.9 ± 0.02
Ti-QAL	Glutaraldehyde	0.7 ± 0.22
Ti-QAHSPro	Glutaraldehyde	−
Ti-QAH-Pro_ML	Multi-layering Glutaraldehyde	0.4 ± 0.20
Ti-QAH_Cyan	HDMI	4.5 ± 1.20
Ti-QAL_Cyan	HDMI	1.0 ± 0.81

**Table 4:** EDS elementary analysis of Ti-QAH compared to untreated Ti and oxidized Ti (Mass Normalized %).

Element	Ti	Ti-Ox	Ti-QAH
Carbon	1.5	1.5	25.2
Oxygen	3.9	8.9	25.5
Titanium	94.6	89.6	43.5
Silicium			3.1
Natrium			2.3

## Supplementary Materials

Supplementary materials can be found at https://www.mdpi.com/1422-0067/20/24/6297/s1.

## Abbreviations

APTES	3-aminopropyltrietoxysilane
CFU	Colony forming unit
Ch	Chitosan
CSH	High molecular weight Chitosan
CSL	Low molecular weight Chitosan
CV	Crystal violet
DEAE	2-diethylaminoethyl chloride
EOFM	Electroosmotic flow mapping
FE-SEM	Field Emission-Scanning Electron Microscopy
FPA	Focal plane array
HACC	N-2-hydroxypropyltrimethylammonium Chitosan
HDMI	Hexamethylene diidocyanate
MBCs	Minimal Bactericidal concentrations
MHB	Mueller Hinton broth
MICs	Minimal Inhibitory concentrations
6-MNA	6-mercaptonicotinamide 6-MNA
MW	Molecular weight
PIA	Polysaccharide intercellular adhesin
PS	Physiological solution
QAH	High molecular weight quaternized chitosan
QAH-CD	High molecular weight quaternized chitosan grafted with methyl-β-cyclodextrin
QAH-Pro	High molecular weight quaternized chitosan with pendant protected thiols
QAL	Low molecular weight quaternized chitosan
QAL-CD	Low molecular weight quaternized chitosan grafted with methyl-β-cyclodextrin
QAL-Pro	Low molecular weight quaternized chitosan with pendant protected thiols
SEC	Size exclusion cromatography
SEM	Scanning electron microscopy
TEA	triethylamine
Ti	Untreated titanium
Ti-OX	Titanium subjected to etching
Ti-QAH	Titanium surface grafted with QAH
Ti-QAHcyan	Titanium surface grafted with QAH by using hexamethylene diisocyanate as crosslinker
Ti-QAH-Pro	Titanium surface grafted with QAH-Pro
Ti-QAH-Pro_ML	Titanium surface grafted with QAH-Pro by multi-layer grafting approach
Ti-QAL	Titanium surface grafted with QAL
Ti-QALcyan	Titanium surface grafted with QAL by using hexamethylene diisocyanate as crosslinker
TSA	Tryptone Soy agar
TSB	Tryptone Soy broth
