# Correction: Piras, A.M., et al. Antibacterial, Antibiofilm, and Antiadhesive Properties of Different Quaternized Chitosan Derivatives. *Int. J. Mol. Sci.* 2019, *20*, 6297

**DOI:** 10.3390/ijms22052511

**Published:** 2021-03-03

**Authors:** Anna Maria Piras, Semih Esin, Arianna Benedetti, Giuseppantonio Maisetta, Angela Fabiano, Ylenia Zambito, Giovanna Batoni

**Affiliations:** 1 Department of Pharmacy, University of Pisa, 56126 Pisa, Italy; angela.fabiano@unipi.it (A.F.); ylenia.zambito@unipi.it (Y.Z.); 2 Department of Translational Research and New Technologies in Medicine and Surgery, University of Pisa, 56126 Pisa, Italy; semih.esin@med.unipi.it (S.E.); a.benedetti16@studenti.unipi.it (A.B.); gmaisetta@biomed.unipi.it (G.M.); 3 Interdepartmental Research Center Nutraceuticals and Food for Health, University of Pisa, 56126 Pisa, Italy

The authors wish to make the following corrections to this paper [1]:

The third column of Table 1 was erroneously named Deacetylation Degree (DD, %), whereas it is obviously referring to the Acetylation Degree (AD, %) of the chitosan derivatives, as clearly discussed in the text of the manuscript. Table 1 summarizes the main characteristics of the polymers, giving an immediate picture of the investigated biomaterials. For this reason, the authors have deemed it necessary to provide a corrected version of the Table 1 heading. The original table is to be replaced with the following one. 

The authors would like to apologize for any inconvenience caused to the readers by these changes.

## Figures and Tables

**Table 1:** Main chemical characteristics of precursors (CSH and CSL) and Ch derivatives.

Polymers	MW, kDa; A_2_	AD ^a^, %	QD ^b^, %; n	TD ^c^, %	DSD ^d^, %	PD ^e^, %	CDD ^f^, %
CSH	379; 0.0009	-	-		-	-	-
CSL	131; 0.0008	-	-	-	-	-	-
QAH	428; 0.0001	7	85; 2	-	-	-	-
QAL	163; 0.0013	3	80; 2	-	-	-	-
QAH-Pro	460; 0.0001	7	85; 2	1.1	17.0	3.1	-
QAL-Pro	161; 0.0007	3	80; 2	1.2	14.9	2.3	-
QAH-CD	2344; 0.0001	7	85; 2	-	-	-	16
QAL-CD	633; 0.0001	3	80; 2	-	-	-	10

^a^ Acetylation degree; ^b^ quaternization degree; ^c^ thiolation degree; ^d^ disulfide degree; ^e^ protection with 6-mercaptonicotinamide (6-MNA) degree; ^f^ degree of substitution with methyl-β-cyclodextrin.
