# Supplementary material for: Antibacterial, Antibiofilm, and Antiadhesive Properties of Different Quaternized Chitosan Derivatives
Source: Int J Mol Sci. 2019 Dec 13;20(24):6297. doi: 10.3390/ijms20246297 (PMC6940869; doi:10.3390/ijms20246297)
Supplement: Supplementary file 1 [file ijms-20-06297-s001.pdf]

Supplementary materials.

Antibacterial, antibiofilm, and antiadhesive properties of different quaternized chitosan derivatives

Anna Maria Piras*, Semih Esin, Arianna Benedetti, Giuseppantonio Maisetta, Angela Fabiano, Ylenia Zambito, and Giovanna Batoni*

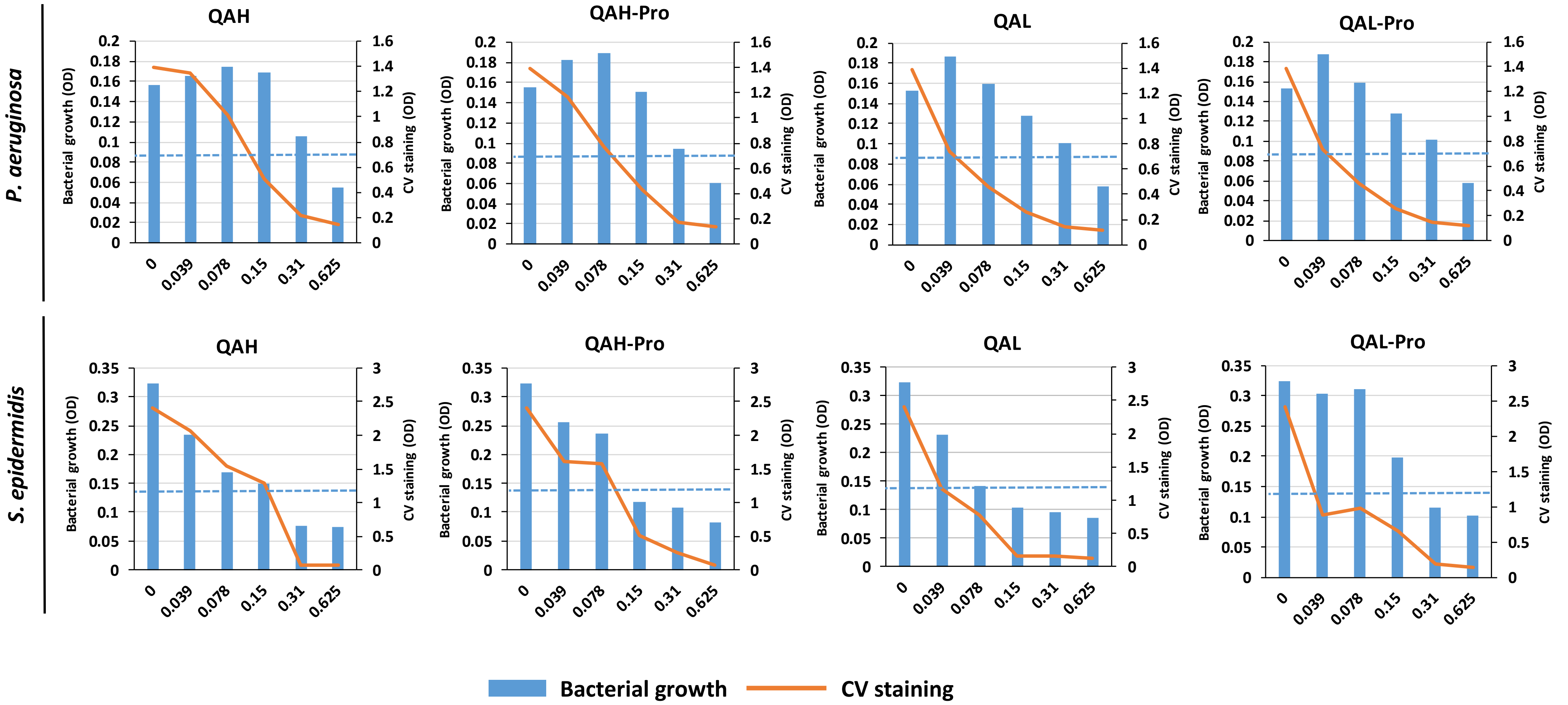

Supplementary Figure S1: Relationship between bacterial growth in the supernatant and biofilm formed at the bottom of the wells. Stationary phase cultures of *P. aeruginosa* or *S. epidermidis* were distributed in wells of 96 well-plates in the presence of different concentrations of chitosan derivatives (mg/ml). Following overnight incubation, bacterial growth in the supernatant was quantified by OD at 590 nm (blue bars). After washing of the wells to remove non-adhered cells, biofilm at the bottom of the wells was quantified by CV staining (orange lines). The Figure depicts the results of a representative experiment.

FUNCTIONALIZATION OF TITANIUM SURFACE

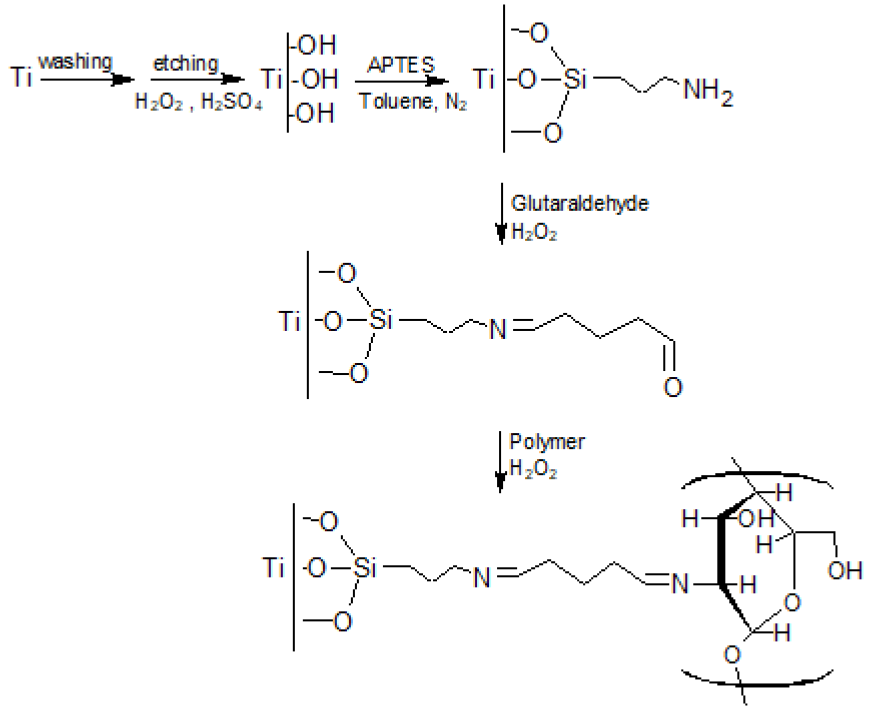

Scheme S1. Stages of polymer grafting on the titanium surface: Step1: washing; Step 2: etching; Step 3: silanization; Step 4: linker insertion (e.g. glutaraldehyde), Step 5: polymer grafting.

ATTENUATED TOTAL REFLECTANCE FOURIER TRANSFORM INFRARED SPECTROSCOPY

The titanium surfaces were also characterized by microscopy coupled to infrared (Cary 660 series FT-IR, Agilent Technologies). Spectra were acquired in attenuated total reflectance (ATR), in percent transmittance, in a range between 500 cm^{-1} and 4000 cm^{-1} with a resolution interval of 4 cm^{-1} and a scanning number of 1000. The spectrum of titanium subjected to etching was subtracted from the acquired spectra of titanium samples.

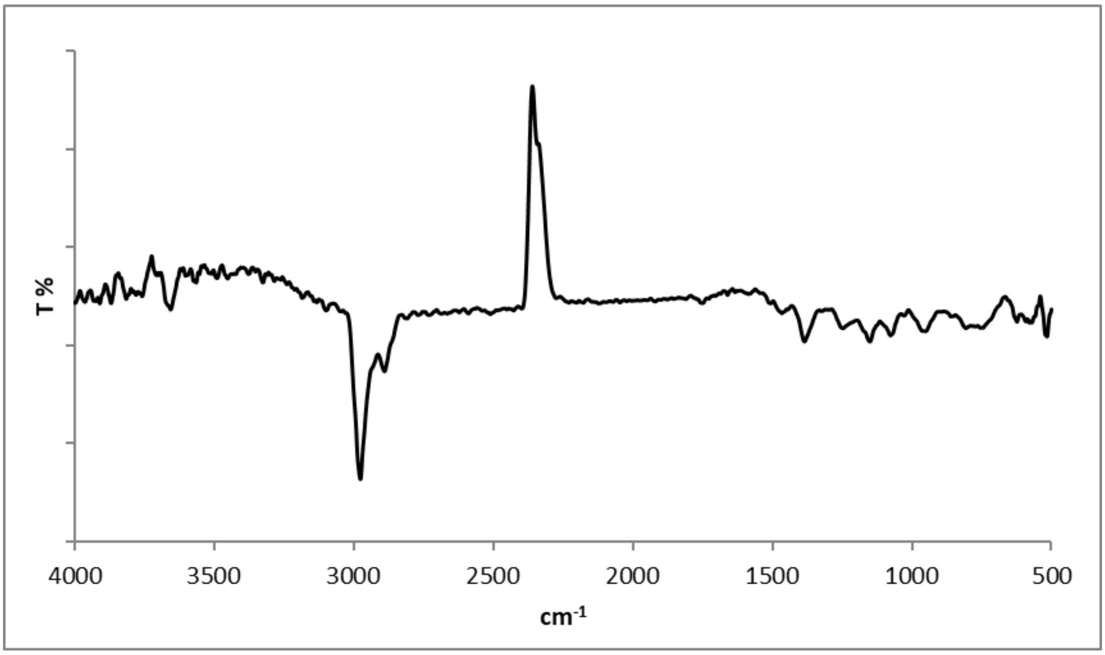

Figure S2. ATR/FT-IR of silanized titanium surface (Ti-APTES): 2890 e 2984 (ν CH_2 of APTES), 1590 (bending $-NH_2$), 1246 (ν C-N), 1150 (ν Si-O-Si), 957 (ν Si-OH).

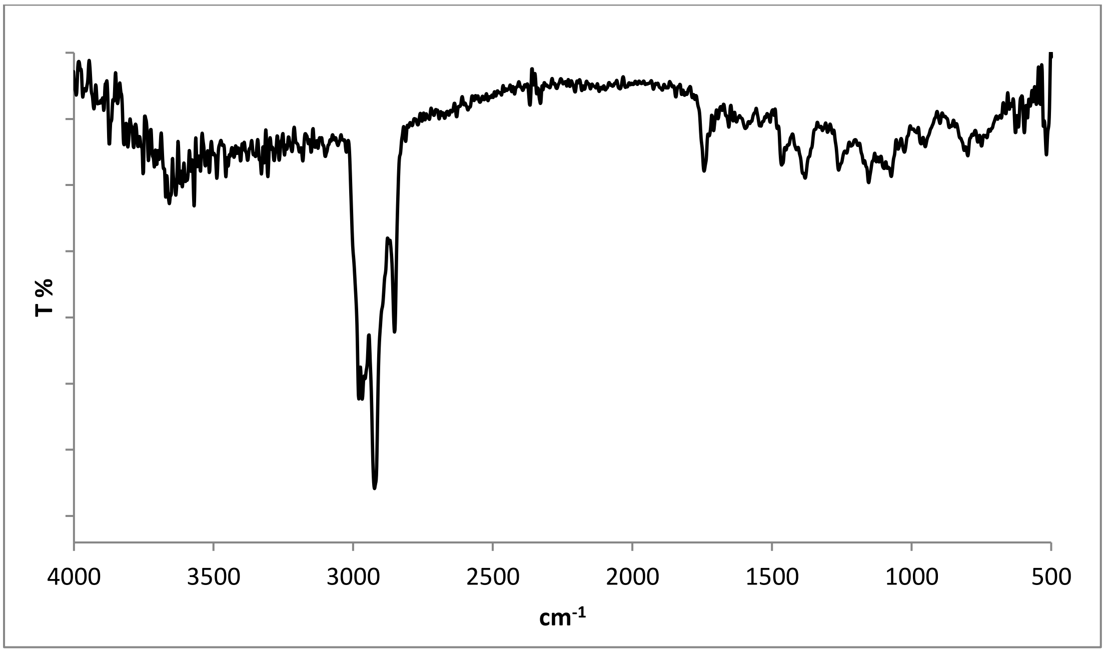

Figure S3. ATR/FT-IR of glutaraldehyde activated titanium surface (Ti-Glu): 2927 (ν CH_2), 2825 & 2720 (ν H-CO), 1722 (ν HC=O), 1457 (bending CH_2), 1376 (bending H-CO).

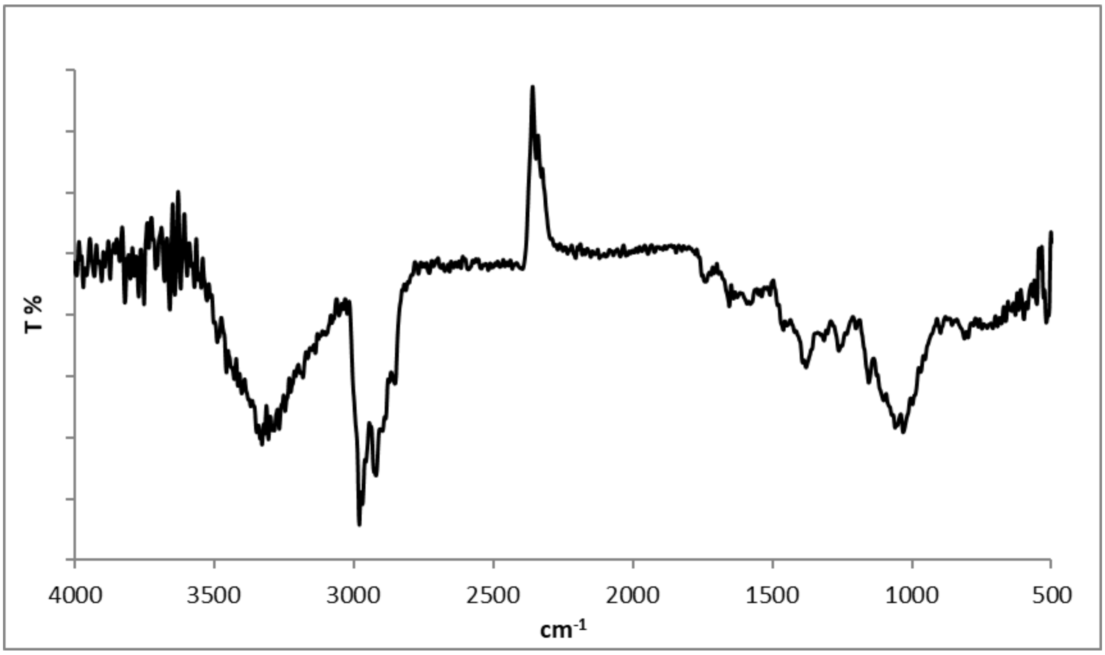

Figure S4 ATR/FT-IR of QAH grafted on Titanium surface (Ti-QAH): 1656 (ν C=O), 1639 (ν C=N), 1590-1564 (bending –NH_2 residue and II amide band), 1480-1456 (bending CH_2 and CH_3), 1392 (bending CH_3), 1380 (bending CH_2), 1317 (ν C-N), 1098 (ν C-N tertiary amine), 1028 (ν C-O-C).

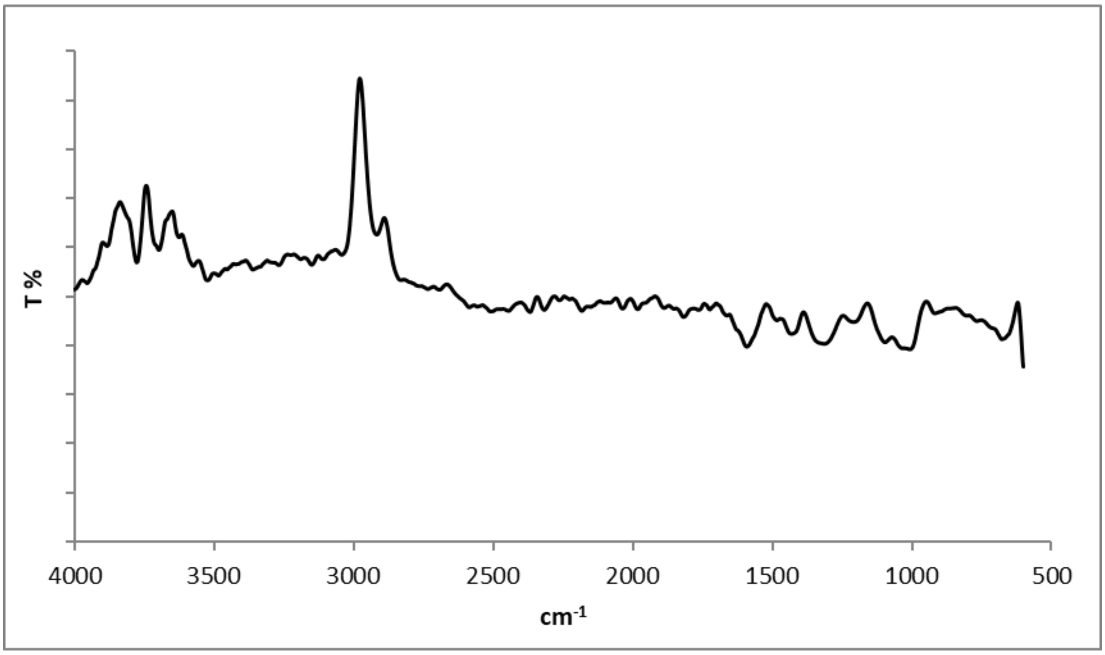

Figure S5. ATR/FT-IR of QAH-Pro grafted on Titanium surface (Ti-QAH-Pro): 1659 (ν C=O), 1636 (ν C=N), 1589 (bending $-NH_2$ residue and II amide band), 1486 and 1368 (bending CH_2 and CH_3), 1320 (ν C-N), 1105 (ν C-N tertiary amine), 1025 (ν C-O-C).

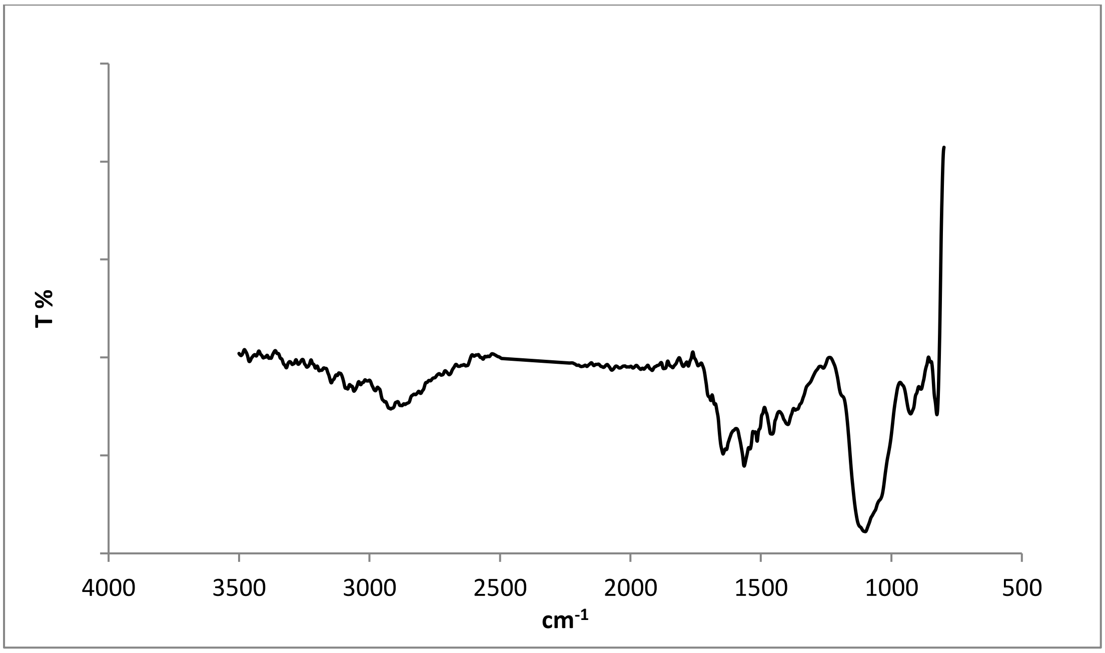

Figure S6 ATR/FT-IR of QAL grafted on Titanium surface (Ti-QAL): 1657 (ν C=O), 1639 (ν C=N), 1590-1564 (bending $-NH_2$ residue and II amide band), 1460 and 1394 (bending CH_2 and CH_3), 1320 & 1246 (ν C-N), 1101 (ν C-N tertiary amine), 1025 (ν C-O-C).

Preliminary screening of silanization by using APTES

Step 3 of the titanium surface functionalization involved the reaction with 3-aminopropyltrietoxysilane (APTES, Sigma-Aldrich). For the setup of functionalization conditions, ethanol, ethanol/HCl 0.05 mM 33:1, or ethanol/H2O (1:1), or toluene were arrayed. Each titanium surface previously etched was immersed in 5 mL of a 5% (v/v) APTES solution (in either ethanol, ethanol/HCl, ethanol/H2O, or toluene) and the reaction was proceeded for 24 h in a shaker water bath

(25 °C, 80 rpm). In case of ethanol and toluene, the reaction proceeded under N_2 atmosphere. Next, the samples were washed with 20 mL each of reaction solvents, followed by ethanol and deionized water; dried by wiping with filter paper, then under vacuum. Samples thermal curing was performed by heating to 110 °C in an oil bath for 20 min under an N_2 stream.

Table S1 reports the quantification of amine moieties on titanium surfaces resulting from the use of different solvents; as determined by picric acid method.

Table S1. Dependence of amino group density on solvent used for silanization (mean ± SD) as determined by picric acid assay.

Solvent	Density, nmol/mm^2
Ethanol	$7.7 \cdot 10^{-2} \pm 4 \cdot 10^{-2}$
Ethanol/H_2O	$4.4 \cdot 10^{-2} \pm 4 \cdot 10^{-3}$
Etahanol + HCl 0.5 mM	$5.8 \cdot 10^{-2} \pm 2 \cdot 10^{-3}$
Toluene	$59.2 \cdot 10^{-2} \pm 3 \cdot 10^{-2}$

Method of multilayering conjugation of QAH-Pro on titanium surface – Ti-QAH-Pro-ML

For multilayering grafting of QAH-Pro on titanium surface, stages 4 and 5 of the functionalization protocol were repeated 4 times. In details, silanized titanium samples were immersed in 5% glutaraldehyde solutions in 50 mM pH 7.5 phosphate buffer for 4 h at 25 °C, washed three times by dipping in 20 mL of deionized water in sequence and wiped dry by capillarity with filter paper, subsequently transferred to aqueous solutions (pH 7) of polymers (0.5mg/mL) and incubated 24 h at 25 °C. This procedure was repeated 4 times, finally the samples were washed twice with 20 mL of deionized water, wiped dry with filter paper and vacuum dried at 37 °C.

Method of Grafting on Titanium surfaces by using HMDI as spacer (Ti-QAH-Cyan and Ti-QAL-Cyan)

As an alternative to glutaraldehyde, hexamethylene diisocyanate (HMDI) was used as a longer crosslinker. Silanized titanium surfaces were dried under vacuum, 700 µL of DMSO, 100 µL of HMDI, and 10 µL of triethylamine (TEA) were added under N_2 atmosphere and kept for 24 h. Freshly distilled HMDI, DMSO and TEA were used. Following incubation, the samples were washed with DMSO then transferred to 1mL of polymer solution (0.5 mg/mL in DMSO, 1% TEA) and left for 24 h at 25°C. Subsequently, they were abundantly washed with DMSO, deionized water, ethanol and ether.

<u>CHARACTERIZATION OF POLYMERS</u>

^{1}H-NMR

NMR measurements of quaternized and cyclodextrin grafted polymers were carried out with a Bruker Avance II operating at 250.13 MHz. During data acquisition the temperature was kept at 25 ± 0.1 °C. For analysis the samples were dissolved in D_2O (1-2 %). The acquisition of ^{1}H-NMR spectra, shown in Figures S7-S10, allowed for the determination of quaternarization degree and cyclodextrin conjugation fractions. The proton unit of the quaternized chitosan was determined from the area under the signals at 4.2-2.2 ppm, corresponding to the protons of the chitosan repeating unit, but for the anomeric one, and the methylene of the pendant chain. In addition, the following were calculated: deacetylation degree, from the diagnostic acetyl groups, at 2.0 ppm; quaternization degree, in terms of quaternary ammonium moieties per repeating unit; and mean length of pendant chains, from the signals of methyl end groups at 1.6-0.7 ppm. Concerning the functionalization with cyclodextrin, the signal at 2.5 ppm of the terminal methylene groups of the quaternized pendant was used as reference and the signals of the cyclodextrin anomeric protons at 5.2 and 5.0 ppm as diagnostic.

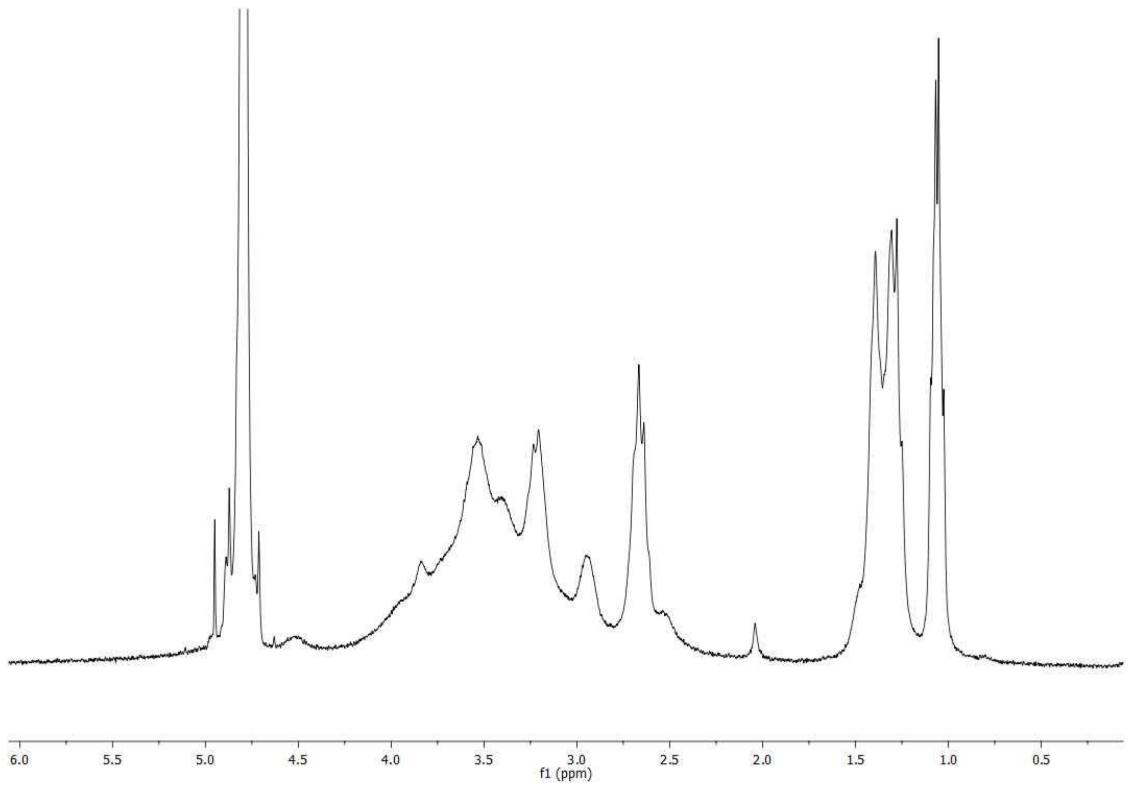

Figure S7: ^{1}H-NMR of QAH: δ = 4.5 (s, anomeric), 4.2-2.2 (m, pyranosidic ring, and methylene protons of pendant quaternized chains $–C\mathbf{H_2}C\mathbf{H_2}N^+(C\mathbf{H_2}CH_3)_2C\mathbf{H_2}$- and $–C\mathbf{H_2}N(C\mathbf{H_2}CH_3)_2$), 2.0 (s, methyl protons of *N*-acetylglucosamine), 1.6-1.1 (m, methyl protons of the ethyl moieties closed to ammonium pendants $N^+CH_2C\mathbf{H_3}$), 1.1-0.8 ppm (m, methyl protons of the terminal part of DEAE chains $-N(CH_2C\mathbf{H_3})_2$).

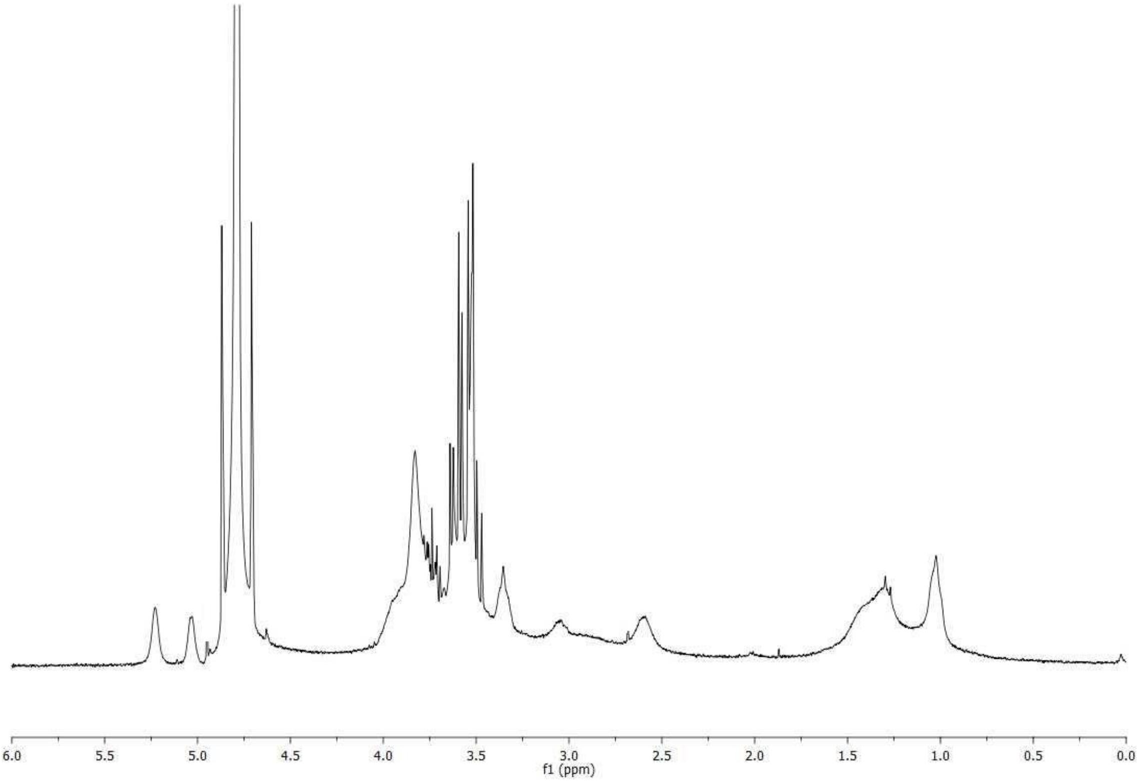

Figure S8: ^{1}H-NMR of QAH-CD. δ = 5.13 and 4.95 (s, MDC anomerics), 4.2–2.6 (m, protons of the pyranosidic ring, methylene protons of pendant quaternized chains $–C\mathbf{H_2}C\mathbf{H_2}N^+(C\mathbf{H_2}CH_3)_2C\mathbf{H_2}$- and $–C\mathbf{H_2}N(CH_2CH_3)_2$, methylene protons closed to the N of the spacer $–NHC\mathbf{H_2}(CH_2)_4C\mathbf{H_2}NH_2$); 2.5 (s, methylene protons of not protonated N of the DEAE pendant $–CH_2N(C\mathbf{H_2}CH_3)_2$), 2.0 (s, methyl protons of N-acetylglucosamine), 1.7–1.1 (m, methyl protons of the ethyl moieties closed to ammonium pendants $N+(CH_2C\mathbf{H_3})_2$ and methylene protons of the central part of spacer chain $-NHCH_2(C\mathbf{H_2})_4CH_2NH_2$), 1.0-0.7 ppm (m, methyl protons of the terminal part of DEAE chains $–N(CH_2C\mathbf{H_3})_2$).

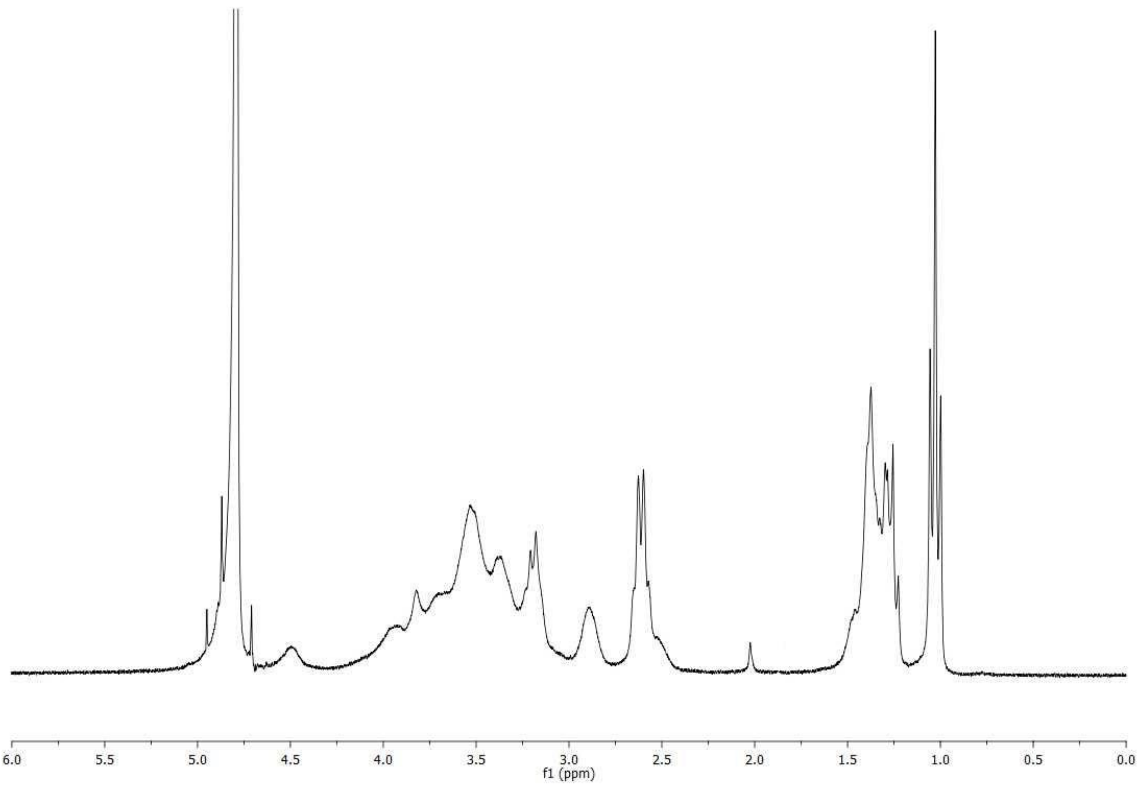

Figure S9: ^{1}H-NMR of QAL. δ = 4.5 (s, anomeric), 4.2-2.2 (m, pyranosidic ring, and methylene protons of pendant quaternized chains –$\mathbf{CH_2CH_2}N^+(\mathbf{CH_2}CH_3)_2\mathbf{CH_2}$- and –$\mathbf{CH_2}N(\mathbf{CH_2}CH_3)_2$), 2.0 (s, methyl protons of *N*-acetylglucosamine), 1.6-1.1 (m, methyl protons of the ethyl moieties closed to ammonium pendants $N^+CH_2\mathbf{CH_3}$), 1.1-0.8 ppm (m, methyl protons of the terminal part of DEAE chains -$N(CH_2\mathbf{CH_3})_2$).

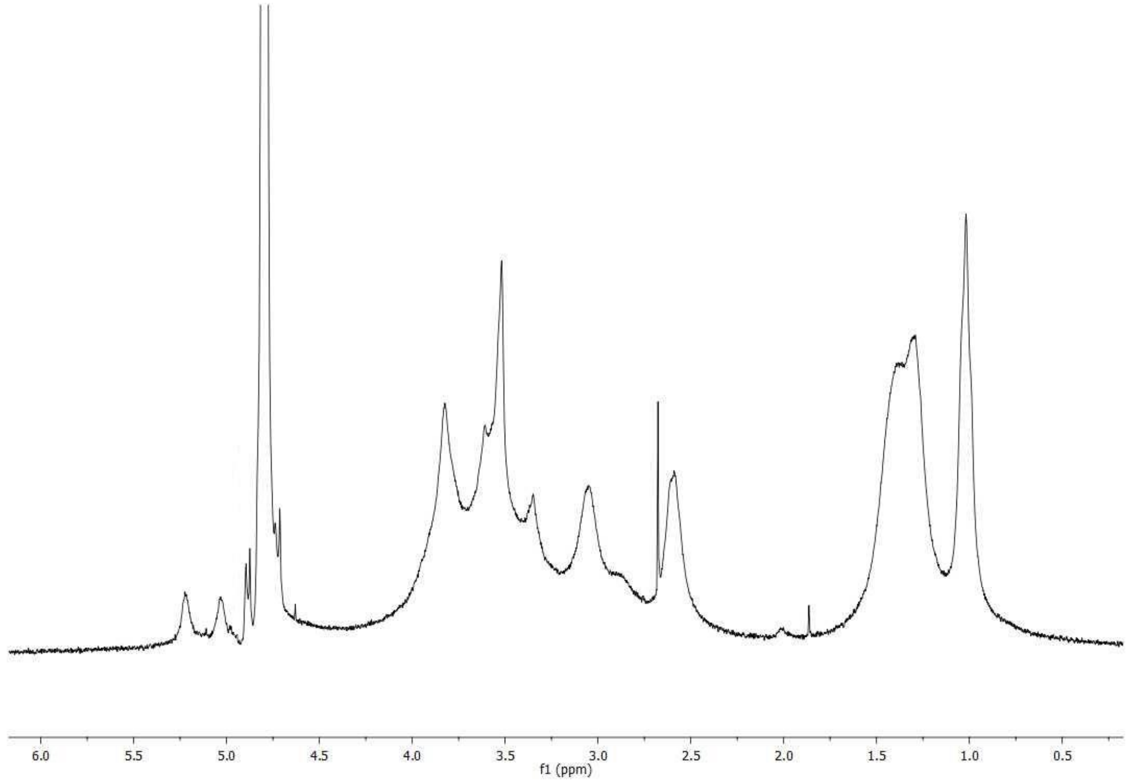

Figure S10: ^{1}H-NMR of QAL-CD. δ = 5.13 and 4.95 (s, MDC anomerics), 4.2–2.6 (m, protons of the pyranosidic ring, methylene protons of pendant quaternized chains –$\mathbf{CH_2CH_2}N^+(\mathbf{CH_2}CH_3)_2\mathbf{CH_2}$- and –$\mathbf{CH_2}N(CH_2CH_3)_2$, methylene protons closed to the N of the spacer –$NH\mathbf{CH_2}(CH_2)_4\mathbf{CH_2}NH_2$); 2.5 (s, methylene protons of not protonated N of the DEAE pendant –$CH_2N(\mathbf{CH_2}CH_3)_2$), 2.0 (s, methyl protons of N-acetylglucosamine), 1.7–1.1 (m, methyl protons of the ethyl moieties closed to ammonium pendants N+$(CH_2\mathbf{CH_3})_2$ and methylene protons of the central part of spacer chain -$NHCH_2(\mathbf{CH_2})_4CH_2NH_2$), 1.0-0.7 ppm (m, methyl protons of the terminal part of DEAE chains –$N(CH_2\mathbf{CH_3})_2$).

Method of thiol determination and protection

The quantification of free thiol moieties was performed for both QAH-Pro and QAL-Pro. Polymers were dissolved in 10 mL of water, 1% starch aqueous solution (1 mL) was added, the pH was adjusted to 3with 1 M HCl and the solution was titrated with 1 mM aqueous iodine until a permanent light blue discoloration was observed. The quantification of degree of substitution by thiol-bearing groups in both polymers was performed after borohydride red-ox reaction. In details, 8 mL of 10% aqueous sodium borohydride was added to a solution of 15 mg polymer in 2 mL of water, and stirred for 1 h. Then the excess of sodium borohydride was destroyed by adjusting to pH 3 with1 M HCl and the thiol content was determined by iodometric titration. The quantification of the protected thiols was performed on samples of 0.1% polymer solution (5 mL), reduced with glutathione (0.1%; 2 h), then analyzed spectrophotometrically at 307 nm. A calibration curve was obtained with 6-MNA (6-mercaptonicotinamide) standard solutions. The content of conjugated aromatic ligand was expressed as the percent of degree of substitution on polymer repeating units.

Molecular weight determination by DEBYE Plot

Polymers Mw was evaluated by building a Debye Plot for each polymer on static light scattering on Litesizer tool, Anton Paar with laser λ658nm scattering angle 90 °. The Debye Plot was constructed by analyzing the variation of scattering intensity with the variation of polymer solution concentration. The weight average molecular weight was calculated according to the relation $KC / R_a = 1 / M + 2A_2C$, where K is an optical constant, Ra is the Rayleigh ratio, M is the weight average molecular weight, A_2 is the virial coefficient and C is the concentration of the polymer in solution. Clear polymer solutions, 0.22μm filtered, were prepared by dissolving the polymers into 0.25 M acetate buffer pH 4.5. The analyzes were carried out in quartz cuvettes, setting the analysis temperature at 25 °C, with toluene used as a reference. The differential increase value of the refractive index as a function of concentration (dn/dc) was set at 0.192 mL/g for plain chitosans [1] and calculated on the basis of size exclusion chromatography linear regressions for chitosan derivatives, according to the procedure adopted for polymer grafting quantification.

References

1. Nguyen S., Winnik F. M., Buschmann M. D., (2009). Improved reproducibility in the determination of the molecular weight of chitosan by analytical size exclusion chromatography. Carbohydrate Polymers, 75: 528–533.
